# Supplementary material for: The effects of geographical distributions of buildings and roads on the spatiotemporal spread of canine rabies: An individual-based modeling study
Source: PLoS Negl Trop Dis. 2022 May 10;16(5):e0010397. doi: 10.1371/journal.pntd.0010397 (PMC9126089; doi:10.1371/journal.pntd.0010397)
Supplement: S1 Text — (PDF) [file pntd.0010397.s001.pdf]

Supporting Information

The effects of geographical distributions of buildings and roads on the spatiotemporal spread of canine rabies: An individual-based modeling study

Chayanin Sararat[1], Suttikiat Changruenngam[1], Arun Chumkaeo[2], Anuwat Wiratsudakul[3], Wirichada Pan-ngum[4], and Charin Modchang[1,5*]

[1] Biophysics Group, Department of Physics, Faculty of Science, Mahidol University, Bangkok Thailand.

[2] Songkhla Provincial Livestock Office, Muang, Songkhla, Thailand

[3] Department of Clinical Sciences and Public Health, and the Monitoring and Surveillance Center for Zoonotic Diseases in Wildlife and Exotic Animals, Faculty of Veterinary Science, Mahidol University, Nakhon Pathom, Thailand

[4] Department of Tropical Hygiene, Faculty of Tropical Medicine, Mahidol University, Bangkok, Thailand.

S1 Text: Derivation of the encounter rate

Assuming that dog movement is a random process, the dispersal of a dog at a particular location (x, y) around their home location (x_0, y_0) is governed by the circular Gaussian function:

$$P(x,y) = a \exp\left(\left(\frac{(x-x_0)^2+(y-y_0)^2}{2d_0^2}\right)\right),$$

where d_0 denotes the mean traveling distance of the dog. Considering dog i and dog j residing a distance d_{ij} apart and assuming that the home location of dog i is located at a position (0,0), the illustration of home locations of these two dogs are shown in **Figure S1**.

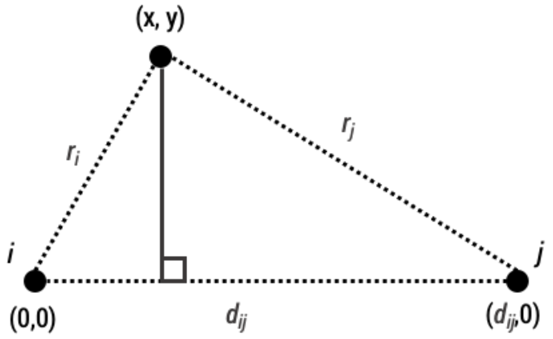

Figure S1: Illustration of home location of the dog i and the dog j

Thus, the probability density of finding the dog i at the location (x, y) is

$$P_i(x,y) = a_i\, exp\left(-\frac{x^2+y^2}{2d_{0,i}^2}\right),$$

and likewise, the probability density of finding the dog j at the location (x, y) is

$$P_j(x,y) = a_j\, exp\left(-\frac{(x-d_{ij})^2+y^2}{2d_{0,j}^2}\right).$$

Multiplication of the $P_i(x, y)$ and $P_j(x, y)$ is a representative of the encounter probability density of the dog i and the dog j at the location (x, y). The integral over space of the unnormalized encounter probability density corresponds to the encounter probability of the dog pair at a particular time, which is

$$P_{ij} = \int_0^T \iint_{-\infty}^{\infty} P_i(x, y) P_j(x, y)\, dxdydt.$$

$$P_{ij} = T \iint_{-\infty}^{\infty} a_{ij} \exp\left[-\left(\frac{x^2 + y^2}{2d_{0,i}^2}\right)\right] \exp\left[-\left(\frac{(x - d_{ij})^2 + y^2}{2d_{0,j}^2}\right)\right] dxdy.$$

$$P_{ij} = Ta_{ij} \iint_{-\infty}^{\infty} \exp\left[-\left(\frac{x^2}{2d_{0,i}^2} + \frac{(x-d_{ij})^2}{2d_{0,j}^2}\right)\right] \exp\left[-\left(\frac{y^2}{2d_{0,i}^2} + \frac{y^2}{2d_{0,j}^2}\right)\right] dxdy.$$

$$P_{ij} = Ta_{ij} \int_{-\infty}^{\infty} \exp\left[-\left(\frac{x^2}{2d_{0,i}^2} + \frac{(x-d_{ij})^2}{2d_{0,j}^2}\right)\right] dx \int_{-\infty}^{\infty} \exp\left[-\left(\frac{y^2}{2d_{0,i}^2} + \frac{y^2}{2d_{0,j}^2}\right)\right] dy.$$

$$P_{ij} = Ta_{ij} \int_{-\infty}^{\infty} \exp\left[-\left(\frac{1}{2d_{0,i}^2} + \frac{1}{2d_{0,j}^2}\right)x^2 + \left(\frac{d_{ij}}{d_{0,j}^2}\right)x + \left(-\frac{d_{ij}^2}{d_{0,j}^2}\right)\right] dx \int_{-\infty}^{\infty} \exp\left[-\left(\frac{1}{2d_{0,i}^2} + \frac{1}{2d_{0,j}^2}\right)y^2\right] dy.$$

Applying the following integration of an arbitrary Gaussian function,

$$\int_{-\infty}^{\infty} e^{-a(x+b)^2}\, dx = \sqrt{\frac{\pi}{a}},$$

And the alternative form,

$$\int_{-\infty}^{\infty} e^{-ax^2+bx+c}\, dx = \sqrt{\frac{\pi}{a}}\, e^{\frac{b^4}{4a}+c},$$

thus,

$$P_{ij} = Ta_{ij}\pi \left(\frac{2d_{0,i}^2 d_{0,j}^2}{d_{0,i}^2 + d_{0,j}^2}\right) \exp\left[\left(\frac{d_{ij}^2}{d_{0,j}^4} \times \frac{d_{0,i}^2 d_{0,j}^2}{2(d_{0,i}^2 + d_{0,j}^2)}\right) - \frac{d_{ij}^2}{2d_{0,j}^2}\right].$$

$$P_{ij} = A \exp\left[-\frac{1}{2} \frac{d_{ij}^2}{\left(d_{0,i}^2 + d_{0,j}^2\right)}\right].$$

We assumed that, within a day, dogs living in the same location (same resident) have certainly faced each other. In other words, if d_{ij} = 0 the encounter probability becomes one, P_{ij} = 1. Therefore, we obtain the unnormalized encounter rate between a pair of dogs living a distance d_{ij} apart,

$$K_{ij} = \exp\left(-\frac{1}{2}\frac{d_{ij}^2}{\left(d_{0,i}^2+d_{0,j}^2\right)}\right).$$
