# Supplementary figures and images for: The effects of geographical distributions of buildings and roads on the spatiotemporal spread of canine rabies: An individual-based modeling study
Source: PLoS Negl Trop Dis. 2022 May 10;16(5):e0010397. doi: 10.1371/journal.pntd.0010397 (PMC9126089; doi:10.1371/journal.pntd.0010397)

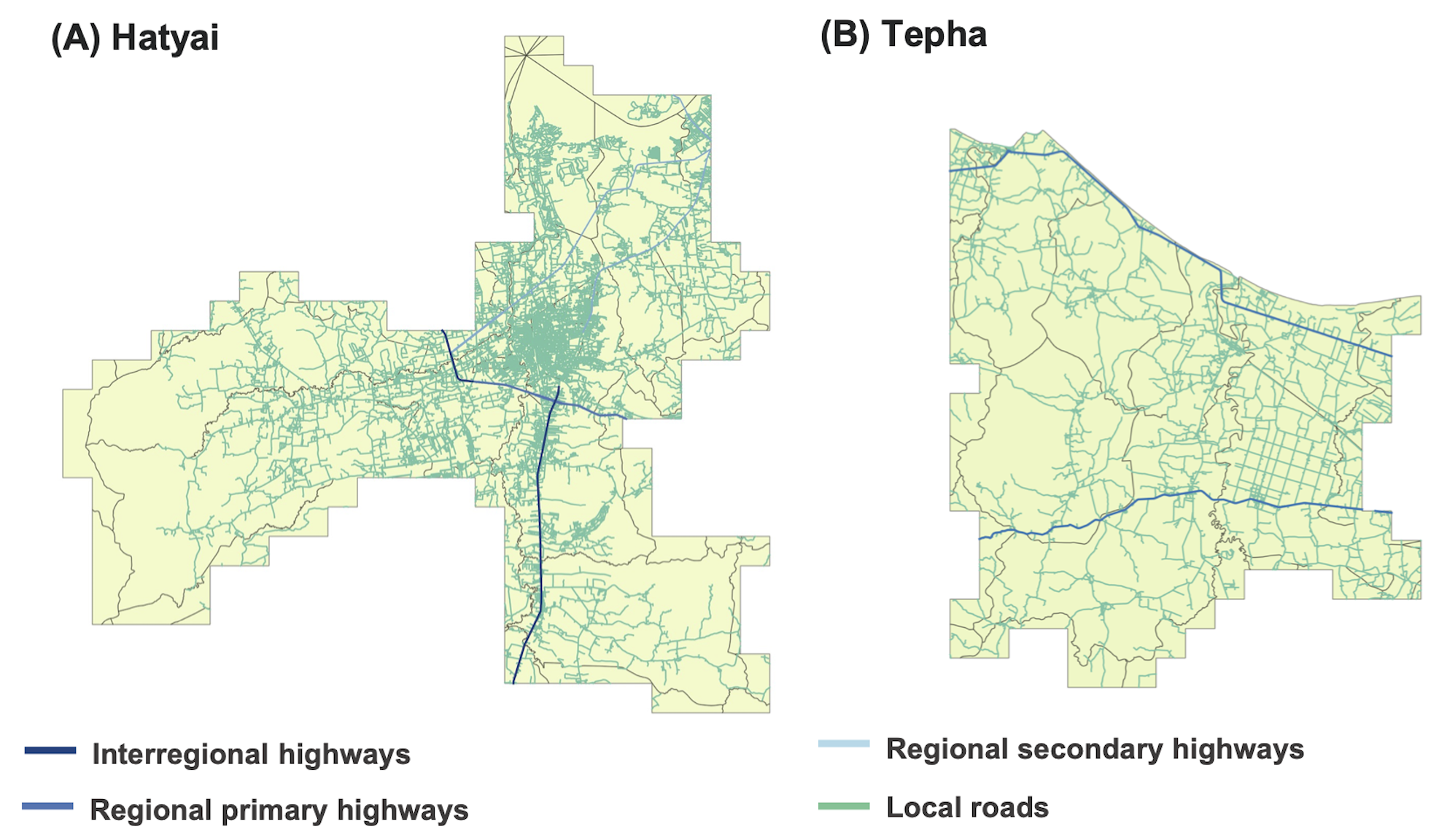

Supplement: S1 Fig — Road alignments in (A) Hatyai and (B) Tepha. In Thailand, roads are typically divided into 4 types, interregional highways, regional primary highways, regional secondary highways, and rural and local roads. Interregional highways are highways connecting Bangkok to outlying regions (for example, Route 4 to southern Thailand). Regional highways are highways within a region. Local roads are roads connecting main roads to important locations. The local roads are usually small roads where traffics is usually not heavy. Therefore, dogs can usually cross and walk along the roads. In the Hatyai area, there is 25.19 km of interregional highways, 15.12 km of regional primary highways, 53.74 km of regional secondary highways, and 2,708.52 km of local roads, representing 0.9%, 0.5%, 1.9%, and 96.7%, respectively. In Tepha, there are no interregional highways; the total length of regional highways and local roads is 65.08 km (5.4%) and 1,138.81 km (94.6%), respectively. The base layer of the map was obtained from https://data.humdata.org/dataset/thailand-administrative-boundaries. (TIFF) [file pntd.0010397.s002.tiff]

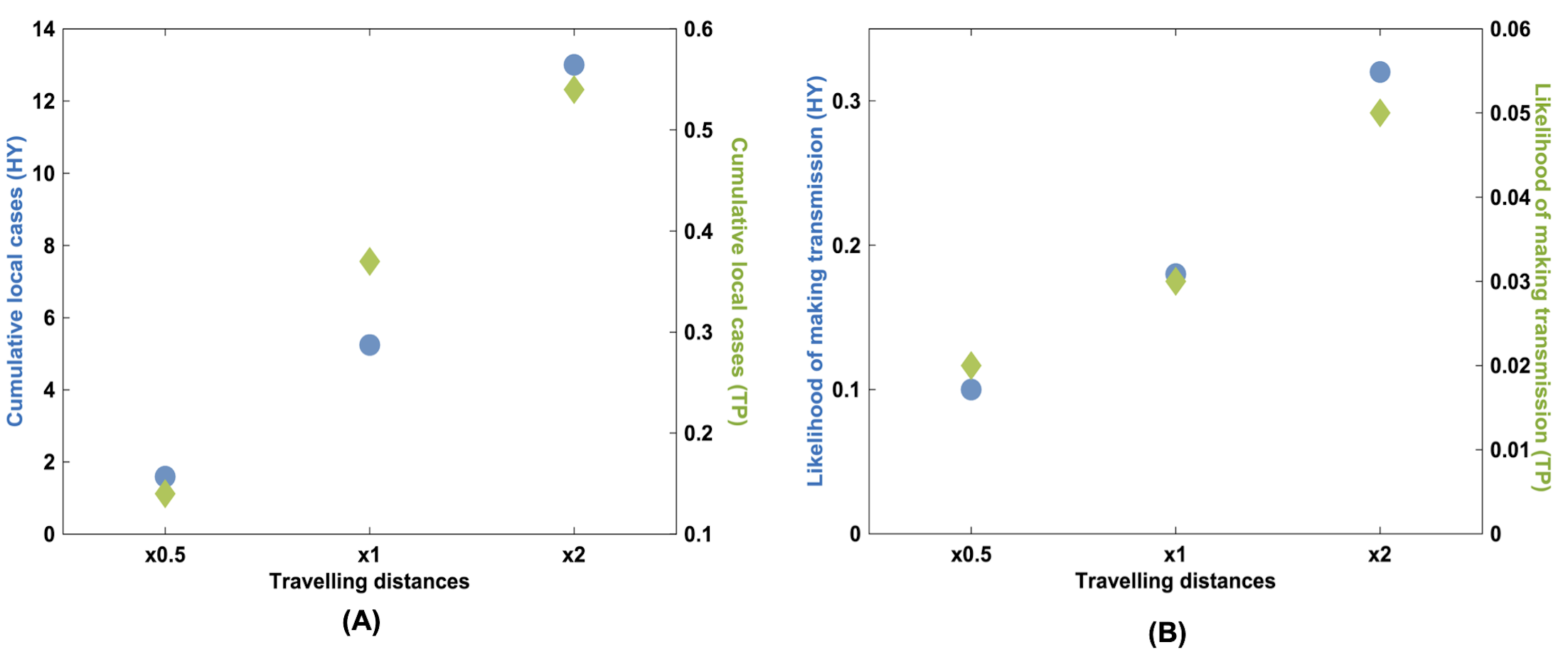

Supplement: S2 Fig — We performed a sensitivity analysis on the dog traveling distances by scaling the mean traveling distances by factors 0.5 (x0.5) and 2 (x2). (A) Cumulative local cases within 365 days of simulations. (B) Likelihood for an imported infected dog to make a secondary infection. Blue and green represent Hatyai and Tepha, respectively. (TIFF) [file pntd.0010397.s003.tiff]

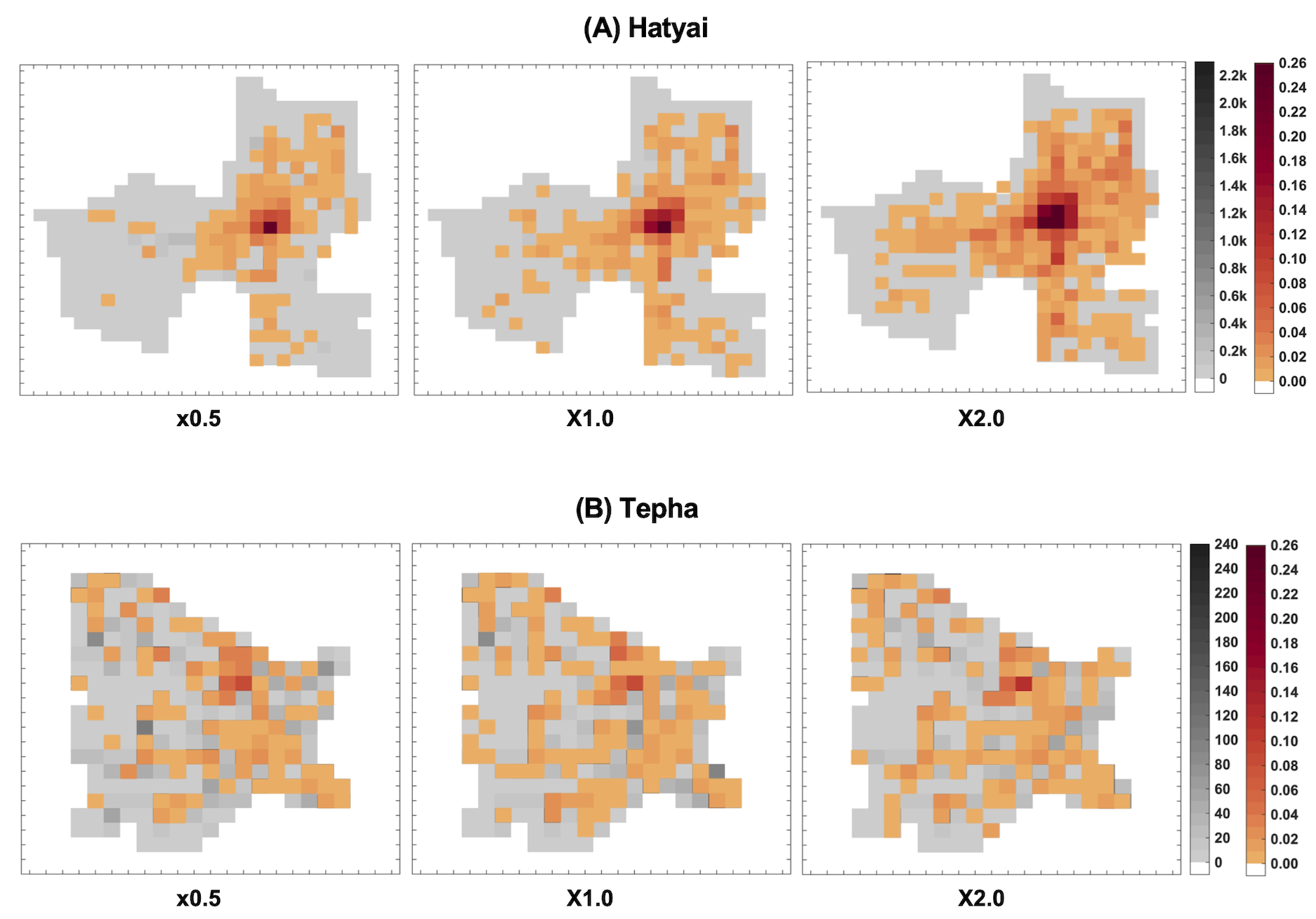

Supplement: S3 Fig — We performed a sensitivity analysis on the dog traveling distances by scaling the mean traveling distances by factors 0.5 (x0.5) and 2 (x2). Each sub-figure depicts the spatial distribution of cumulative rabies cases after one year of rabid dog introduction. The greyscale represents the density of buildings (buildings/km2), while the warm-color scale denotes the cumulative number of rabid dogs (dogs/km2). (TIFF) [file pntd.0010397.s004.tiff]

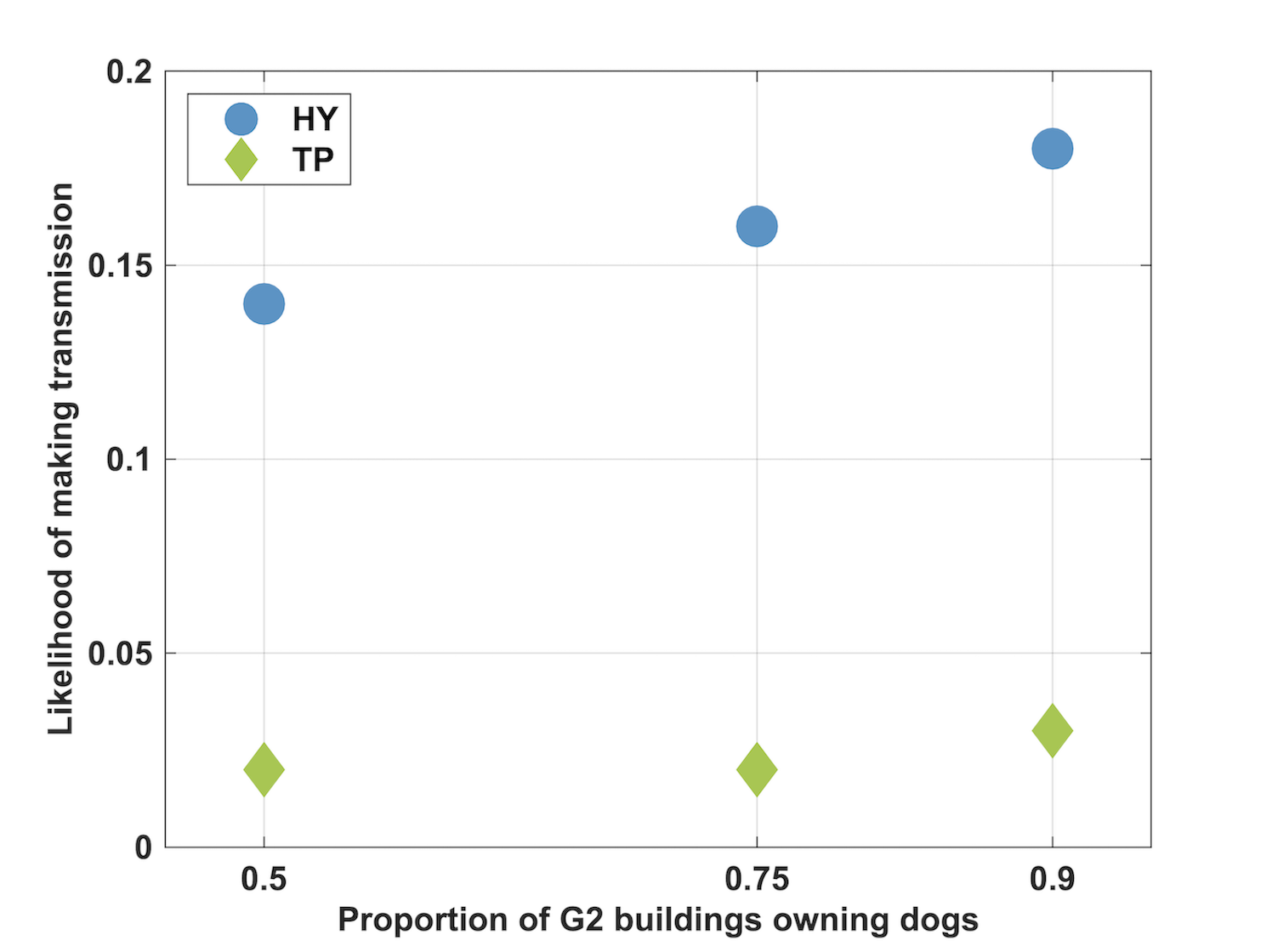

Supplement: S4 Fig — (TIFF) [file pntd.0010397.s005.tiff]

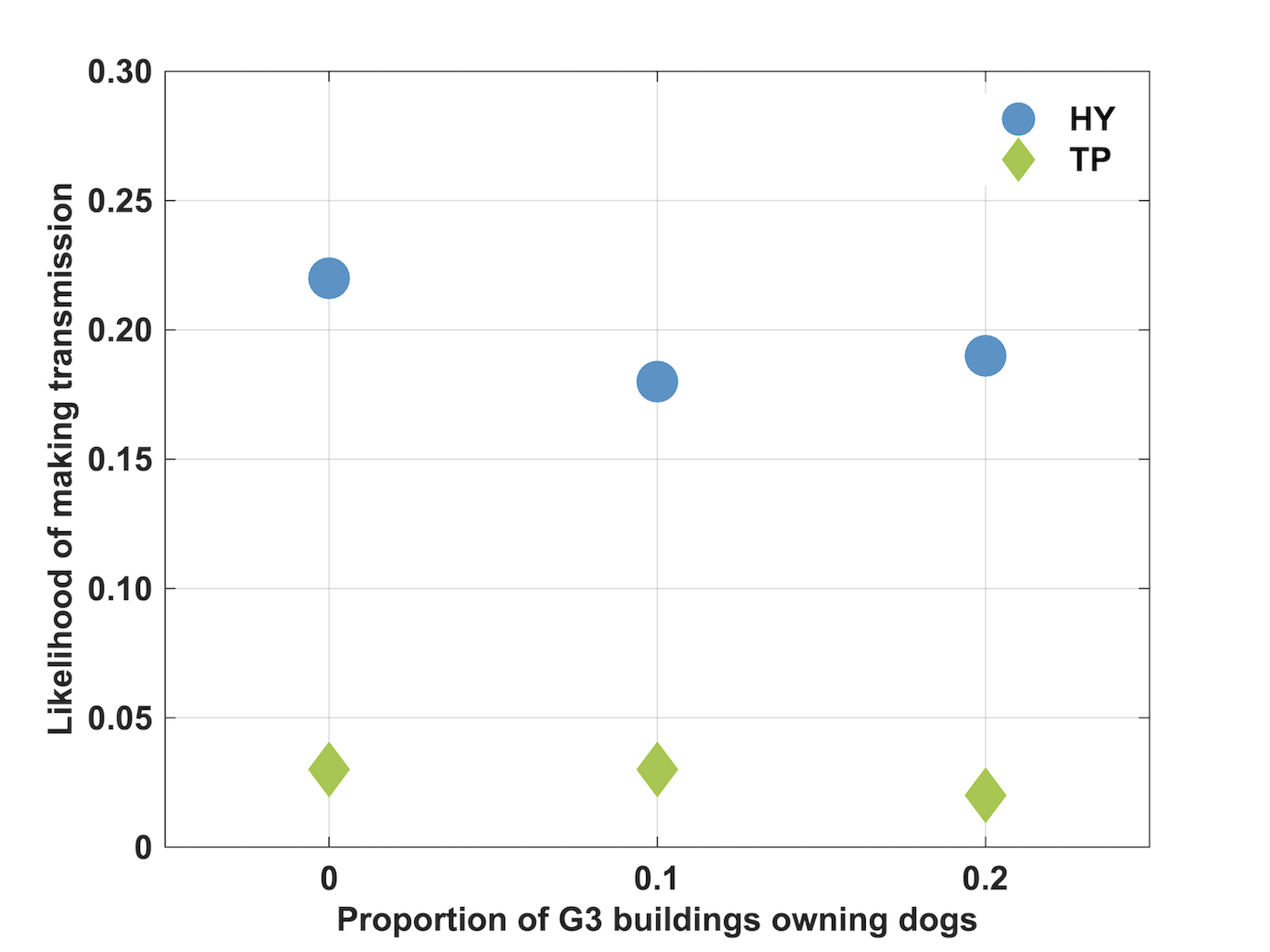

Supplement: S5 Fig — (TIFF) [file pntd.0010397.s006.tiff]

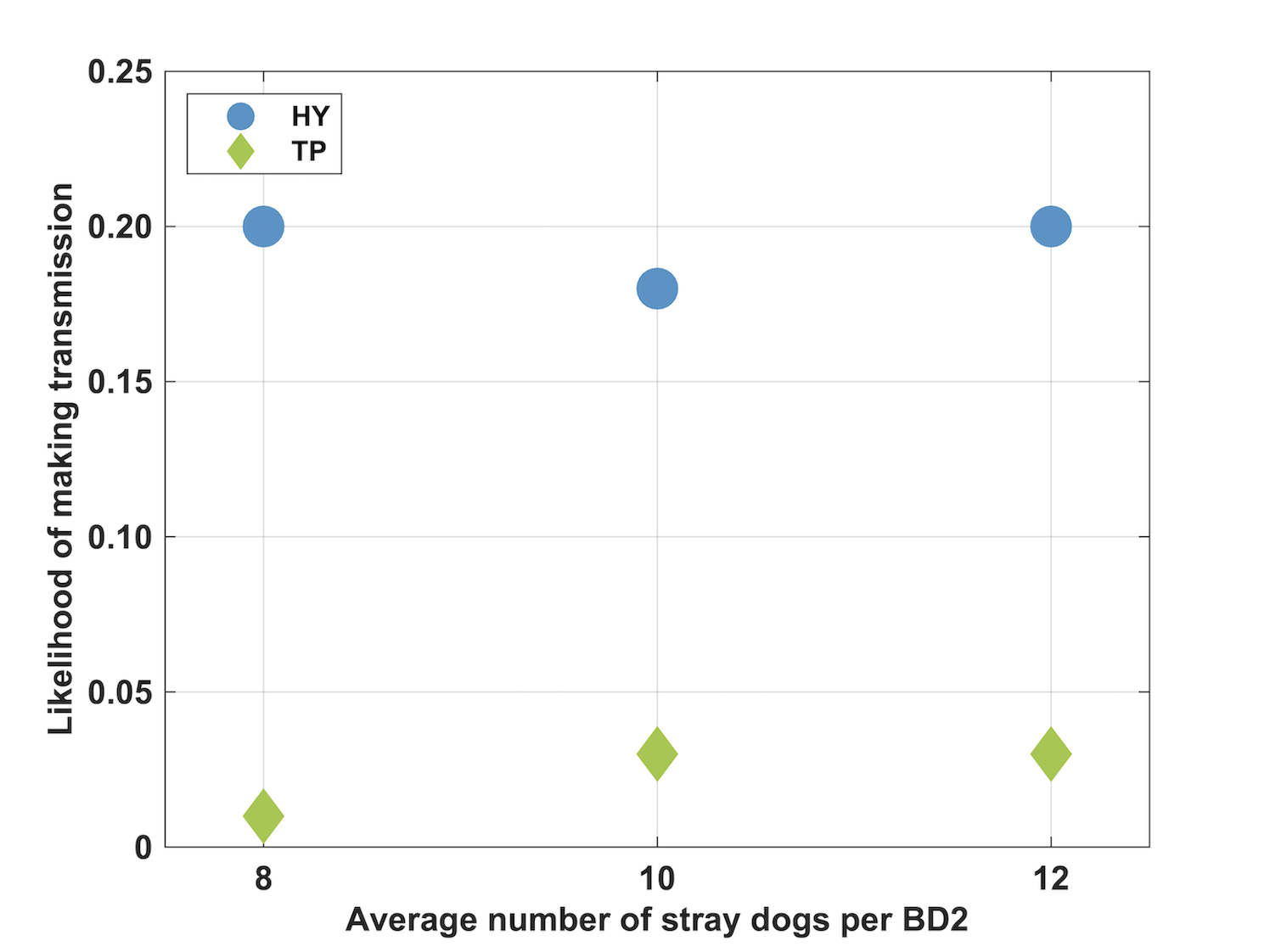

Supplement: S6 Fig — (TIF) [file pntd.0010397.s007.tif]

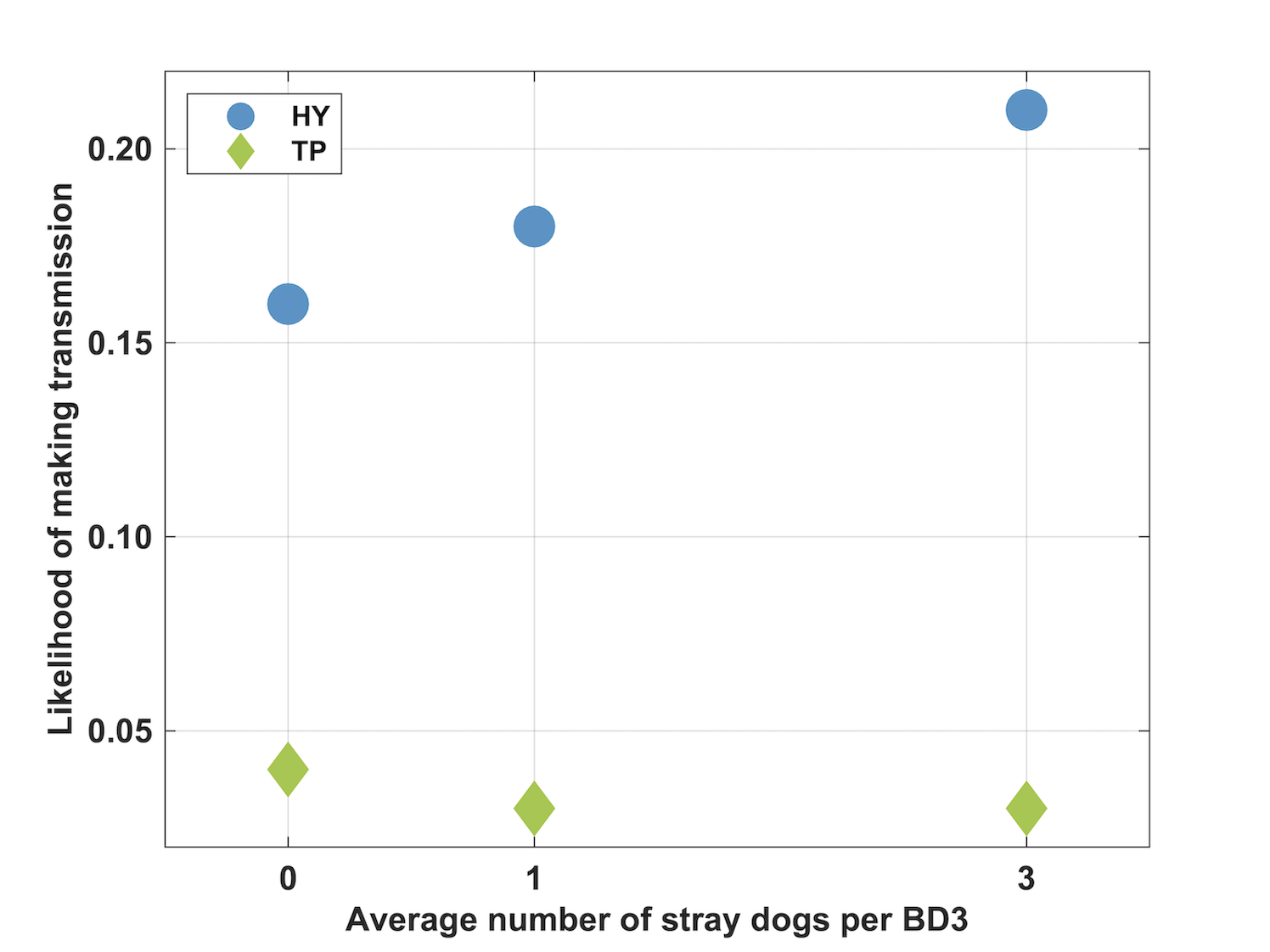

Supplement: S7 Fig — (TIF) [file pntd.0010397.s008.tif]
