# Supplementary material for: The effects of geographical distributions of buildings and roads on the spatiotemporal spread of canine rabies: An individual-based modeling study
Source: PLoS Negl Trop Dis. 2022 May 10;16(5):e0010397. doi: 10.1371/journal.pntd.0010397 (PMC9126089; doi:10.1371/journal.pntd.0010397)
Supplement: Attachment — Submitted filename: Response to referees comments_FINAL.pdf [file pntd.0010397.s011.pdf]

Response to the referee's comments

We would like to thank the referee for their time and effort taken to review our manuscript. The comments from the referee tremendously helped us improve the manuscript. We wish to express our appreciation and hope that we satisfactorily address all the points.

The responses to the referee's comments and all changes made in the main manuscript and the supplement information are listed below and written in **green text** to ease the reading. (Please note that due to the EndNote software citation formatting, a number assigned to a citation reference in this letter is different from those in the revised manuscript.)

Before we describe the point-by-point responses to the referee, we would like to mention that we have substantially revised the manuscript based on the referee's comments. Mainly:

(1) Dog vaccination has been incorporated into the baseline model. As the core structure of the model has been changed, we therefore have entirely reperformed the model calibration and model fitting
(2) References of some model parameters have been updated
(3) Sensitivity analysis for model parameters that data are not available has been performed
(4) Mass vaccination has been included in the study as an intervention strategy

Reviewer #1

Methods

The reviewer stated that "*The authors present a manuscript on the effects of geographical distributions of buildings and roads on the spatiotemporal spread of canine rabies. The authors construct an individual based model or rabies transmission the explicitly considers the built environment and test also non-pharmaceutical interventions like density reduction and confinement of dogs. Although the approach is interesting, the paper is of limited scientific value because of the following reasons:…*"

Comment 1: "*The model is almost exclusively based on assumptions and not on empirical observations (Table 1).*"

Response: We have now updated references of some model parameters in Table 1. Table 1 has been revised, which now reads

Table 1. Parameters and the baseline values used in the rabies transmission model.

Parameters	Values	Reference
Natural birth rate (b)	2.28×10^{-4} day^{-1}	[1]
Mortality rate (m)	$m = bS/N$	-
Importation rate (ε)	0.0082 day^{-1}	estimated based on incidence data
Latency rate (σ)	0.0448 day^{-1}	[2]
Rabies induced death rate (δ)	0.32 day^{-1}	[2]
Vaccination coverage	50.38 %	survey report (unpublished)
Transmission probability (p)	1×10^{-5}	model fitting
Mean travelling distance of a susceptible dog		
- living in a building near roads with confinable fences	0.1 km	assumption
- living in a building near roads without confinable fences	1 km	assumption

- living in a building far from roads with confinable fences	0.05 km	assumption
- living in a building far from roads without confinable fences	0.5 km	assumption
Mean travelling distance of a rabid dog		
- living in a building near roads with confinable fences	0.1 km	assumption
- living in a building near roads without confinable fences	5 km	assumption
- living in a building far from roads with confinable fences	0.05 km	assumption
- living in a building far from roads without confinable fences	2.5 km	assumption
Proportion of household with fence	0.48	[3]
Proportion of G1 buildings that own dogs	0.54	[4]
Proportion of G2 buildings that own dogs	0.9	assumption
Proportion of G3 buildings that own dogs	0.1	assumption
Average number of dogs per dog-owning household	2.67	[3]
Average number of dogs per public place	10	assumption
Average number of dogs per G3 building	1	assumption

For the importation rate, we used the phrase " estimated based on incidence data " instead of "assumption" since the importation rate was estimated using rabies incidence data obtained from the Department of Livestock Development of Thailand. We defined an imported case as the case that occurred after a prior case longer than 26 days (22.3 days of the incubation period [2] plus 3.1 days of the infectious period [2]).

For the proportion of households with fences and the average number of dogs per dog-owning household, the values of these two parameters were acquired from the field observations in Ref. [3]. At the time of this manuscript submission, this reference was not available; however, it has now been published, and the reference for values of these parameters has been added.

In addition, the sensitivity analysis has been performed for the model parameters that empirical data are not available. These parameters include the mean traveling distances of dogs, the proportion of buildings that own stray dogs, and the average number of stray dogs per building owning stray dogs. The results of the sensitivity analysis are shown in Figures S1 – S7 in the supplementary information. The unpublished survey report of vaccination coverage is also provided as an attachment.

Comment 2-1: *"There are several issues with the the model assumptions as outlined below:*

All of the buildings in the city are classified into 3 kinds: (G1) houses/residential, (G2) public places where stray dogs usually can be found, (G3) other types of buildings (e.g., groceries, hotels, banks, etc.). Distance of each building centroid to the closest road is estimated approximately."

Response: We would like to provide more information that we classified buildings into three categories to represent the actual habitat of dogs, particularly in Thailand. To ensure that the estimation of the shortest building-to-road distance is valid, we measured this quantity directly from the GIS maps that we got from the Department of Public Works and Town & Country Planning, Ministry of Interior (DPT) of Thailand. The maps comprise a layer of polygons in which each polygon represents a building as well as a layer of road lines, and each building is labeled with its usage (e.g., school, temple, residence). We calculated centroid points of the buildings to serve as referent points. To depict the roadways, points were generated along the road lines with a 5-meter space. To determine the shortest building-to-road distance, we searched for the nearest point along road lines and then measured the distance between the point and the building's centroid.

Comment 2-2: "*Assumption of dog locations: Owned dogs in G1, 90% of stray dogs in G2, 10% of stray dogs in G3*

** There is no empirical basis shown for such distribution, especially the 90-10 division.*"

Response: We would like to provide more information that in Thailand, it is reasonable to assume that owned dogs live in human's residences. For stray dogs, in the context of Thailand,

they are usually found on the roadways, in front of supermarkets, or in other areas that we have labeled as G3, and a single or a few dogs are seen in a location. However, as most stray dogs gather in some locations with sufficient food, such as fresh markets, schools (leftover food from student lunch), and temples, in a bigger size of clusters than in G3, we separated fresh markets, schools, and temples from the G3 group. Nevertheless, there is no empirical evidence to support the proportions of each building type owning stray dogs (we estimated that 90% of G2 buildings own stray dogs). We acknowledge that this might be a limitation of our study. However, we have performed a sensitivity analysis of these factors. The results are shown in Figures S3 and S4 in the supplementary information.

Comment 2-3: "*Main component of the model is the encounter rate expression, which is simply the distance between two spherical Gaussians. There are several points to note about the implicit assumptions:*

** Such movement is often not Gaussian because of the limitations of man-made structures: the distribution stretches widely along the roads while being very narrow around the buildings.*

** Even if a Gaussian could be fitted for simplicity reasons, that would not be a spherical one, the whole covariance matrix (2x2) must be stated in order to account for the stretching of the distribution. At that point, the derived expression in the paper does not apply.* "

Response: Even though the encounter rate, $K_{ij} = \exp\left(-\frac{1}{2}\frac{d_{ij}^2}{\left(d_{0,i}^2+d_{0,j}^2\right)}\right)$, was derived from spherical Gaussian of a particular dog, it already takes into account the influence of man-made structures through the mean traveling distance parameters, $d_{0,i}$ and $d_{0,j}$. For example, the encounter rate between susceptible dogs in various places at 1 km apart from a rabid dog residing at a centered black point is depicted in the figure below. The blue and green spots on the map show the buildings that near and distant from the roads, respectively. As seen in the box on the right of the figure, although the buildings align in similar radius from a rabid dog's

location, a dog living near roads (blue dot) has a greater possibility to encounter the rabid dog, comparing to dogs living far from roads (green dots).

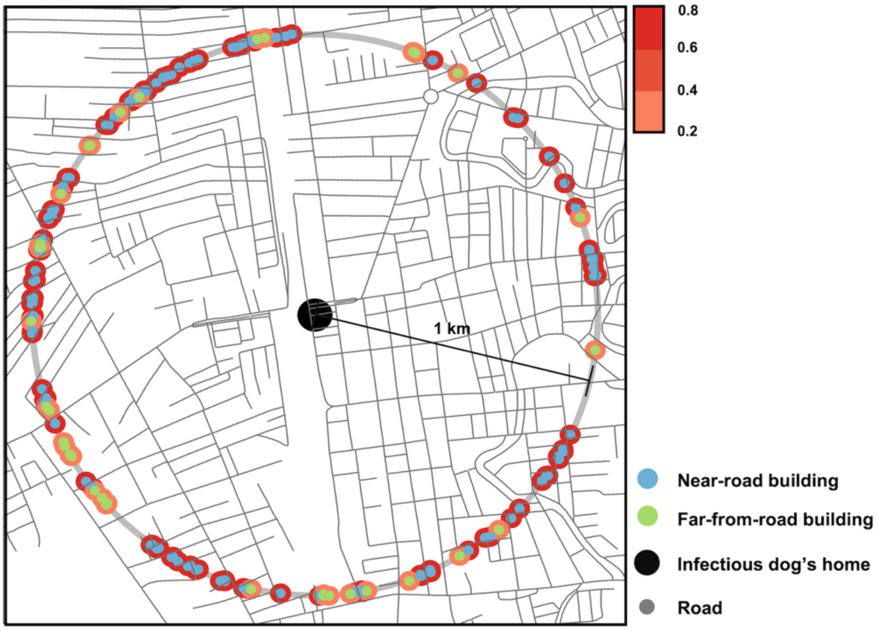

To clarify this point, Fig 1 has been revised, which now reads

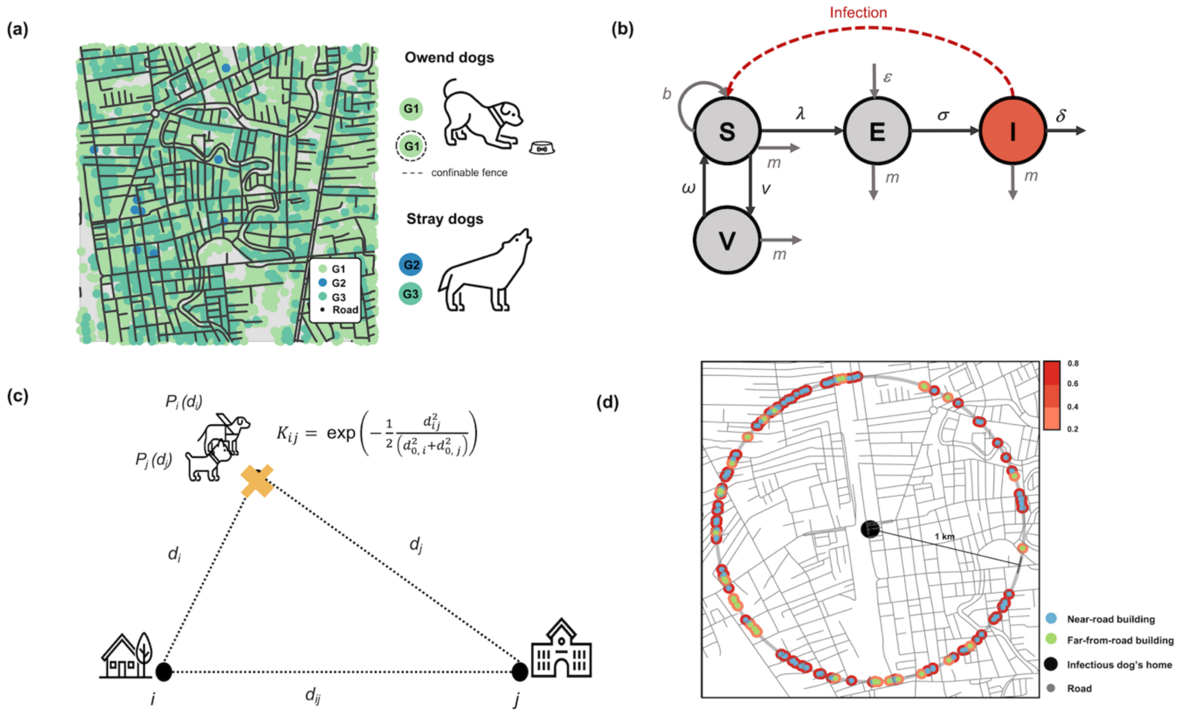

Fig 1. Structure of the canine rabies transmission model. (a) Example of the geographical distribution of buildings and roads in an area of 2x2 km^2. Owned dogs reside only in G1 buildings, while stray dogs may occupy either G2 or G3 buildings. (b) Schematic of the transmission model. Based on the infection status, the model classifies each dog into susceptible (S), exposed (E), infectious (I), and vaccinated (V) classes. The dashed arrow represents transmission events, and the solid arrows indicate transitions between compartments. (c) Illustration of the probability of finding a dog at distance d from their home location, P (d), and the encountering rate between dog i and dog j, K_{ij}. (d) Portray of the encountering rate between a rabid dog resides at the centered black point and susceptible dogs living one kilometer apart. The blue and green dots on the map represent the home locations of susceptible dogs that are located near roads and far from roads, respectively. The encountering rates are indicated by the colors of the dot circumferences.

Comment 2-4: "* *The variances of these distributions (i.e., squared mean traveling distances) are somewhat naively determined: some of the values are taken relatively to the data at hand (e.g., lines 171-173), and nearly all of the values are at the end noted as assumptions in Table 1.*"

Response: We have now performed a sensitivity analysis of the travelling distances (Fig S1 and S2 in the supplementary information) and added the following relevant paragraph in the discussion section:

- However, the main weakness of our study is the absence of empirical data for some model parameters, especially the mean traveling distances of dogs. The roaming ranges of dogs vary greatly depending on the surroundings. For example, the median distance from the residence of free-roaming domestic dogs in a rural area of northern Australia was reported at 63 meters [5] while, in Kenyan rangeland, the values were 309 meters on commercial ranches [6]. Using high-resolution proximity logger to track contact networks of free-ranging domestic dogs in two rural settings in Chad, the total ranges were found to be 20.77 km^2 for Kakale and 4.56 km^2 for Magrao [7], corresponding to circular areas of radius 2.57 km and 4.56 km, respectively. We therefore performed a sensitivity analysis on the dog traveling distances by scaling the mean traveling distances by factors 0.5 and 2. Our simulations demonstrated that longer travel lengths

expand the distance that the disease may spread and result in higher numbers of cumulative cases and more dispersed distributions of cumulative rabies cases (Fig S1 and S2).

Comment 3: "*There is a fundamental shortfall in the consideration of scaling of rabies transmission. We know that dog rabies transmission depends on direct dog to dog transmission on scales of tens of kilometers but also human mediated dog transport on scales of hundreds of kilometers. This interaction is complex and transmission in urban centres often remains endemic only because of continuous importation of rabies, which drive the transmission much more than small scale (< 10 km) spatial effects of the built environment. Unless the authors provide empirical data on the interplay of local transmission between dogs and human mediated dog transport, the analysis of the built environment remains meaningless.*"

Response: We would like to clarify that the human-mediated translocation of dogs was implicitly considered through the importation process in our model. To focus merely on the effect of the distribution difference of buildings and roads, the importation rate was set equally in both areas. Nevertheless, we are aware that importation may be a reason for rabies persistence, and that is a reason why we further considered the likelihood of having a secondary infection if an infected dog was imported.

Results

Comment 1-1: "*The main issue is with the results: there is a circular logic as they design to model to contain relative building-to-road distance as a parameter, and they report that the simulations show different results for cities with different average building-to-road distances.*"

Response: We would like to make it clear that we used the building-to-road distances as a quantity to characterize the architecture structure of our selected sites, specifically to reveal how buildings are distributed around roads (Fig3(a)). Although we then used this quantity to distinguish between near-road buildings and far-from-road buildings, we used the median values of the distribution as a separation line. Consequently, the proportion of near-road buildings is similar in both Hatyai and Tepha. That means, to test our hypothesis, we equalized

all epidemiological parameters as well as dog movements, apart from the geographical distribution of man-made structures in these two areas.

Comment 1-2: "*Furthermore, such simulations, considering the number of hyperparameters (i.e., assumptions) can be very misleading since it is fairly easy to find a set of parameters that show somewhat similar distribution (inside given confidence intervals, as in Fig.4 - a) with the data that consists of a single sample. Yet, if the exact relationship of the distribution and these assumptions cannot be explained, then there is no way to say which ones were the main contributing factors and which ones were trivial.*
As a note, the last sentence is why likelihood maximization over a fixed model is much better than simulation studies, since the factors that contribute to the likelihood are much easier to distinguish than in simulations."

Response: We appreciate the comment from the reviewer. We would like to emphasize that our primary goal is not to provide a model that can accurately describe the rabies transmission in Hatyai and Thepa. Instead, we wanted to investigate if the geographical distribution of buildings could affect the dynamics of rabies transmission. We selected Hatyai and Tepha as study sites because these two locations have contrasting risks of rabies transmission. We have now conducted the sensitivity analysis of some model parameters. We found that across ranges of model parameters, our finding is still robust. Geographical distribution of buildings in Hatyai could facilitate more rabies transmission.

Comment 2: "*The handling of roads seems to be essentially as a passageway for dogs. However from our experience this is highly dependent on traffic. While roads are unambiguous passage ways in low traffic community areas, high traffic roads are important geographical barriers almost interrupting dog-to-dog contact networks1. This is an important shortfall of this paper.*

1. Laager, M. et al. The importance of dog population contact network structures in rabies transmission. PLoS neglected tropical diseases 12, e0006680, doi:10.1371/journal.pntd.0006680 (2018)."

Response: To address this point, we have analyzed the characteristics of roads in our study areas. We found that there are three types of roads in Hatyai and Tepha, namely,

1) Primary highways which are part of the network connecting regions, provinces, districts, and other important destinations
2) Motorways or special highways which are high-capacity highways designed for high-speed traffic
3) Rural and Local highways, which are small local roads.

Alignments of each road type are shown below (and in Figure S8 in the supplementary information). There are 25.19 km of primary highways, 15.12 km of motorways, and 2,762.26 km of local roads in Hatyai, representing 0.9%, 0.54%, and 98.56%, respectively. In Tepha, the total length of motorways and local roads are 65.075 km (5.54%) and 1138.811 km (94.59%), respectively, and there are no primary roads in Tepha. As the majority of roads in these study sites are small local roads in which the traffic is not heavy, we think that the role of the road barrier may be small. To discuss about this point, the following sentences have been added to the last paragraph in the Discussion section:

- Like other modeling studies, there were some limitations in our study. Firstly, while roads show a role to facilitate dog movement [8-13], high-traffic roads, on the other hand, were pointed out to be major geographical obstacles that might disrupt dog roaming [14]. However, as the roads networks in our study sites were dominated by small local roads (98.56% for Hatyai and 94.59% for Tepha) (see Fig S8), we hence did not consider the possible barrier role of roads in our study.

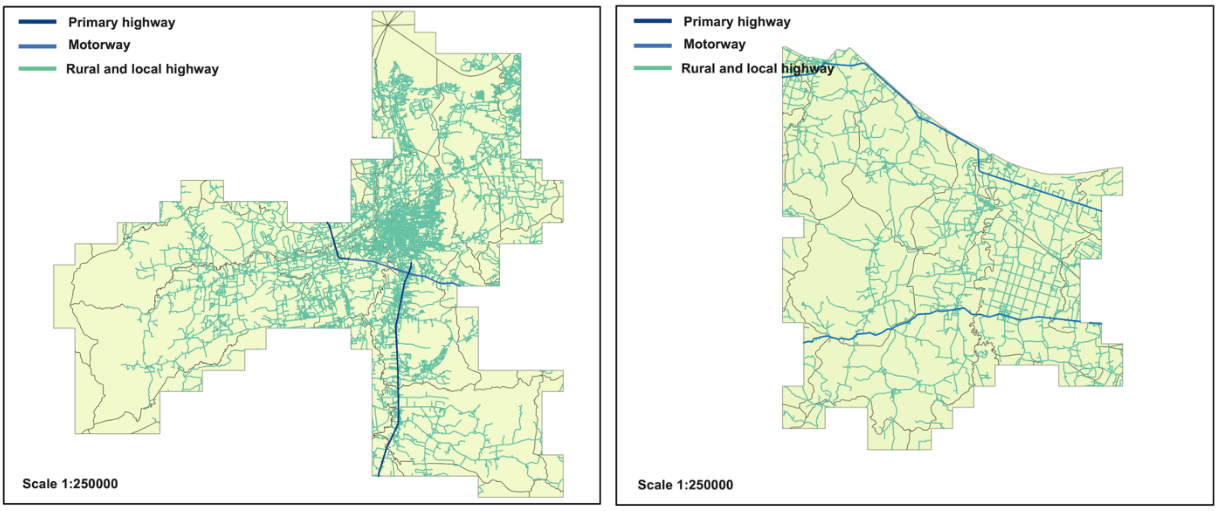

Fig. S8. Road alignments in (a) Hatyai and (b) Tepha. In Thailand, Roads are typically divided into 3 types, primary highways, motorways, and rural and local highways. Primary highways are part of the network connecting regions and provinces. Motorways are high-capacity highways designed for high-speed traffic. Rural and local highways are small local roads where traffics is usually not heavy. Therefore, dogs can usually cross and walk along the rural and local highways roads.

Conclusions

Comment 1: "*The conclusion is a bit disappointing for such an extensive modelling exercise.*

We know since a long time that the reduction of dog density and that confined dogs reduce transmission. This is not new. The reduction of dog density is not a feasible elimination strategy and to confine dogs effectively requires a very high social control. The argument of the authors that the consideration of the distribution of buildings could contribute to target control strategies is not valid. The essential point of effective interventions like mass vaccination of dogs is to reach a sufficiently high coverage over a whole area irrespective of the spatial heterogeneity of the built environment."

Response: We appreciate the referee's comment. We have now entirely revised our study to include the dog vaccination in the model and investigated the mass vaccination of dogs.

The following paragraph has been added to the result section:

- In order to achieve dog-mediated human rabies elimination, mass dog vaccination is an approach recommended by the World Health Organization (WHO). As demonstrated in Fig(c), when dog populations were randomly vaccinated, the likelihood of an imported infected dog causing a secondary infection was lower with the increase of vaccination coverage and eventually disappeared when the whole population was immunized. For instance, in Tepha, vaccinating 80% of the dog population can mitigate the likelihood by three-quarters, and in Hatyai, it cut the risk by more than half.

Figure 6 has been revised, which now reads

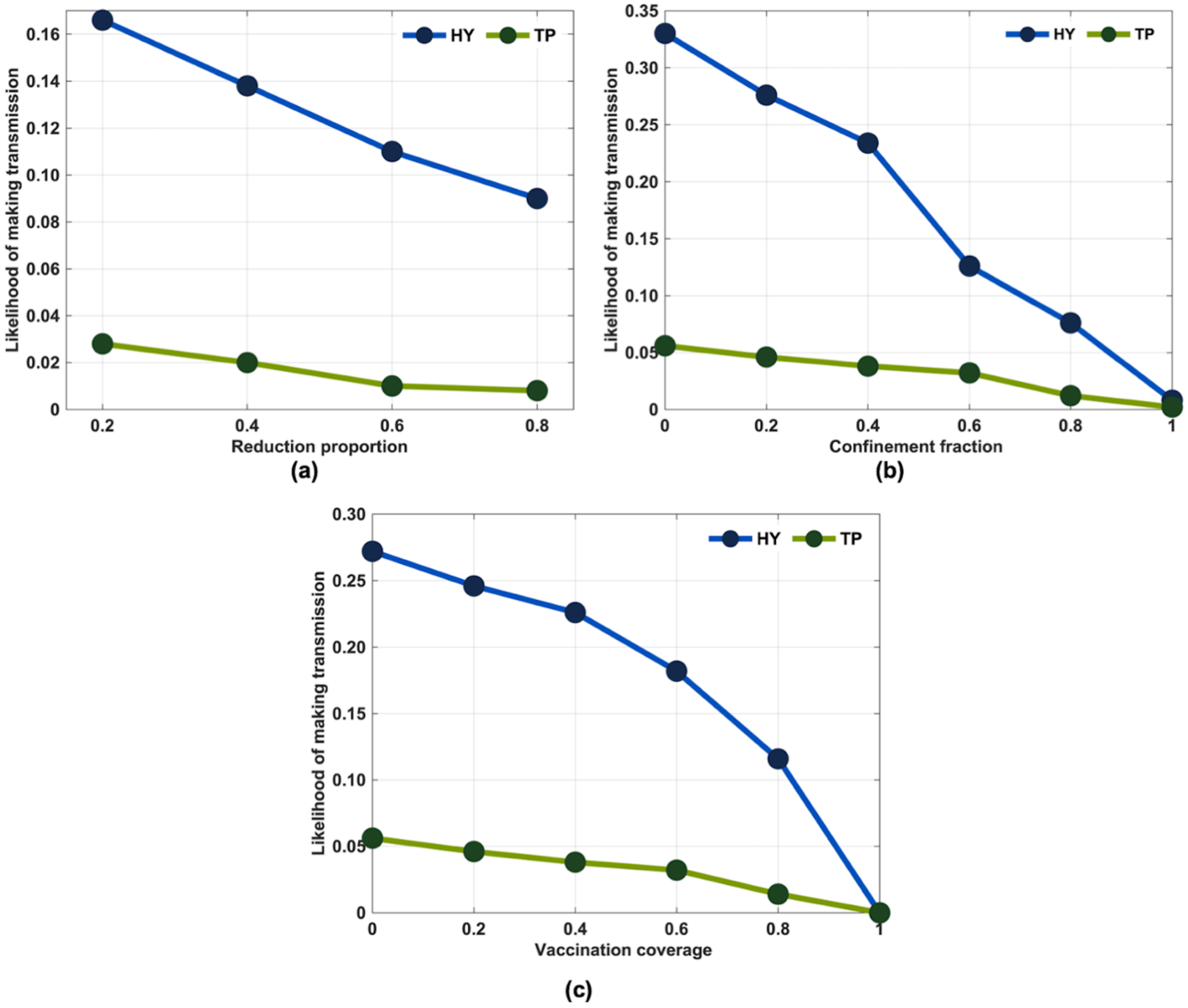

- **Fig 6. Likelihood of rabies transmission caused by an imported infected dog based on different intervention strategies. (a) Population size reduction, (b) owned dog confinement, and (c) mass vaccination.**

The discussion section has been revised to improve the clarity of the conclusion of the study. The following paragraphs have been added to the discussion section:

- In this study, we investigated three intervention strategies, i.e., dog population reduction, dog movement restriction, and mass vaccination. Despite dog density matters for rabies persistence, our results highlighted that reducing the dog population density could diminish the likelihood of rabies transmission. However, if the reduction proportion is inadequately high to clear susceptible dogs within the traveling length of rabid dogs, transmission is still feasible. For example, in Hatyai, where dog density is 243 dogs/km^2, culling half of the population leaves 381 dogs within a one-kilometer roaming radius of a rabid dog. Therefore, it indicated that rabies might not be able to be eliminated by only reducing the dog population. Our finding is in line with a previous study in rural Tanzania where the dog population density is as low as 1.36 dogs per km^2, which is 99% and 97% lower than the dog populations in Hatyai and Tepha district, respectively, canine rabies still circulates in this area [2]. This indicated that implementing only culling strategies might not be effective enough to eliminate rabies [2, 15-18].

- Owned dog confinement also has the potential to decrease the transmission likelihood. Owing to the dense aggregation area in Hatyai, limiting traveling distances of the owned dogs could cut more contacts among dogs than Tepha. We found that restricting owned dog movement could effectively mitigate the transmission in both areas. Moreover, rabies could be eliminated if all owned dogs are restricted inside their homes. Although in the context of Thailand, in which most houses have no fences, strict enforcement on confinement of owned dogs might be impracticable. Therefore, this strategy might need to be implemented in combination with other interventions.

The abstract has also been revised, which now reads

- Rabies is a fatal disease that has been a serious health concern, especially in developing countries. Although rabies is preventable by vaccination, the spread still occurs sporadically in many countries, including Thailand. Geographical structures, habitats, and behaviors of host populations are essential factors that may result in an enormous impact on the mechanism of propagation and persistence of the disease. To investigate the role of geographical structures on the transmission dynamics of canine rabies, we developed a stochastic individual-based model that integrates the exact configuration of buildings and roads. Two contrasting areas with high- and low-risk of rabies transmission in Thailand, namely, Hatyai and Tepha districts, were chosen as study sites. We found that our modeling results generally agree with the reported rabies data. Our results indicated that the distinct geographical structures of buildings and roads in Hatyai and Tepha could contribute to the difference in the rabies transmission dynamics in these two areas. The high density of buildings and roads in Hatyai could facilitate more rabies transmission. We also investigated the impacts of rabies intervention, including reducing the dog population, restricting owned dog movement, and dog vaccination on the spread of canine rabies in these two areas. We found that reducing the dog population alone might not be sufficient for preventing rabies transmission in the high-risk area. Owned dog confinement and dog vaccination could reduce more the likelihood of rabies transmission. These findings are beneficial for the policy improvement to prevent and control canine rabies in Thailand and other countries with similar settings.

Finally, the following sentences have been added to the Author summary:

- We also explored the impacts of reducing dog population, restriction of owned dog movement, and dog vaccination on the rabies spread. Our modeling results indicated that dog vaccination might be a promising approach for achieving the goal of rabies elimination in the high-risk area in Thailand. In addition, different levels of vaccination coverages are probably required to control rabies in different geographical settings.

Summary and General Comments

Comment 1: "*Altogether this paper does not contribute to new knowledge because most of the model parameters are based on assumptions and not on empirical data. The proposal of dog population reduction and total confinement are not feasible. The WHO recommendation of mass vaccination is not addressed by the model. This would have shown that the essential driver of elimination is reaching sufficiently high vaccination coverage, the so called herd immunity.*"

Response: We have now entirely revised our study to include the dog vaccination in the model and investigated the mass vaccination of dogs. We have also revised the discussion and conclusions. Please see the details in response to the comment in the "Conclusions" section.

Reviewer #2:

The reviewer stated that "*The manuscript describes an epidemiological simulation model for rabies spread in dog populations in two provinces in Thailand. The model is based on the geographical distribution of buildings and roads in the two locations and found that the structure influences rabies spread. The authors used the model to simulate the effect of dog density reduction and confinement of owned dogs. The manuscript is well written and can be (mostly) easily followed. Also, it is innovative in the sense that geographical feature of an area is investigated in how this influences rabies spread. However, there are some points that should be improved before the article can be published.*"

Response: We thank the referee for taking time to evaluate our work and for a positive recommendation. We have now revised the manuscript in view of the comments. Please see responses to the comments below.

Main comments:

Comment 1: "*The authors used an approach for rabies incursion into a fully susceptible population (1 dog infectious, all other susceptible). This most likely does not reflect the reality in Thailand, where rabies is endemic since a long time. Most likely, a good proportion of the dog population is not susceptible. It has been mentioned as a limitation of the study that neither vaccination nor other immediate outbreak response were considered in the model. However, the discussion on that aspect needs to be extended. What would be the effect if vaccination were considered? Most likely, this would not be evenly distributed amongst the dog population, but be higher in the owned dogs. Ideally, vaccination is included into the model. This can be done in a simple way by assuming that at the start of the epidemic, x% of the owned dog population is vaccinated.*"

Response: We have revised our study as suggested. The manuscript has been revised throughout to include vaccination of the dog population in the baseline model.

The first paragraph in the model structure section has been revised, which now reads

- We constructed a spatially explicit individual-based model of dog rabies transmission. The model integrates the exact configurations and geographical locations of dogs, buildings, and roads. Dogs are also classified as owned or stray dogs based on the type of building that they live. The model classifies dogs into the following four epidemiological classes: susceptible (*S*), exposed (*E*), infectious (*I*), and vaccinated (*V*) (Fig 1(b)). A susceptible dog can be infected, through a bite of an infectious dog, under the force of infection λ. After being infected, the susceptible dog progresses to the exposed class. Dogs in this class have already acquired the infection but are not yet infectious and cannot transmit the virus to other susceptible dogs. Exposed dogs become infectious at a rate σ, which is inversely proportional to the latent period. Infectious rabid dogs eventually die at a rate δ. All newborn dogs are assumed to be susceptible to the disease and enter the susceptible class at a rate b. To maintain a constant size of the dog population, dogs in all classes were assumed to naturally die at a mortality rate $m = bS(t)/N(t)$. Susceptible dogs are vaccinated at a rate v, while vaccinated dogs lost their vaccination immunity at a rate ω. In order to conserve vaccination coverage of the population, the vaccination rate was set to $\nu = (\omega + m)N_S(t)/N_V(t)$, where $N_S(t)$ and $N_V(t)$ is the total number of susceptible and vaccinated dogs at time t, respectively. In addition to the local transmission, the model also considers the importation of latently infected dogs from surrounding areas, which can occur at a rate ε.

Figure 1(b) has been revised, which now reads

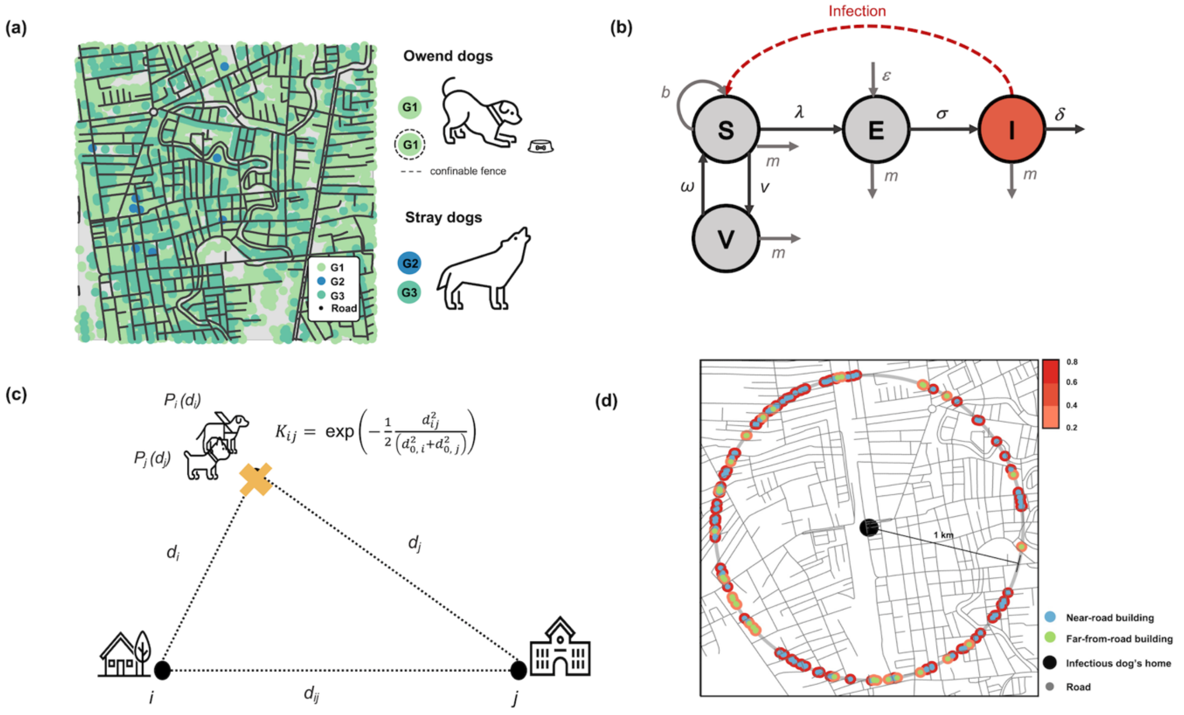

Fig 1. Structure of the canine rabies transmission model. …(b) Schematic of the transmission model. Based on the infection status, the model classifies each dog into susceptible (*S*), exposed (*E*), infectious (*I*), and vaccinated(*V*) classes. The dashed arrow represents transmission events, and the solid arrows indicate transitions between compartments. …

The following paragraph has been added to the result section:

- In order to achieve dog-mediated human rabies elimination, mass dog vaccination is an approach recommended by the World Health Organization (WHO). As demonstrated in Fig(c), when dog populations were randomly vaccinated, the likelihood of an imported infected dog causing a secondary infection was lower with the increase of vaccination coverage and eventually disappeared when the whole population was

immunized. For instance, in Tepha, vaccinating 80% of the dog population can mitigate the likelihood by three-quarters, and in Hatyai, it cut the risk by more than half.

Figure 6 has been revised, which now reads

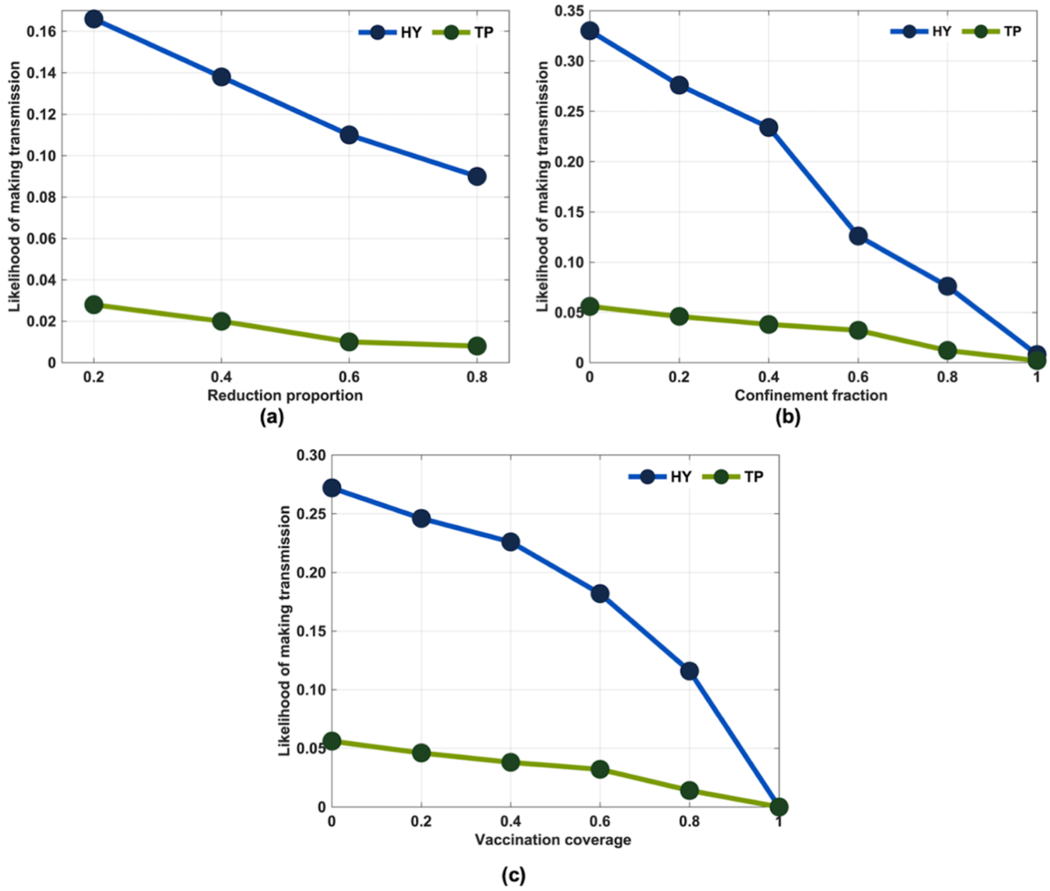

Fig 6. Likelihood of rabies transmission caused by an imported infected dog based on different intervention strategies. (a) Population size reduction, (b) owned dog confinement, and (c) mass vaccination.

Comment 2: "*Related to the former comment, I suggest to extend in the introduction recommended and available rabies control interventions. In my opinion, it needs to be said that vaccination of dogs it the most promising strategy to control the disease. It can be argued that in addition, other control options should also be considered, such as dog population reduction and dog confinement. This is what the authors did in this work.*"

Response: Thank you for pointing this out. We have now added the following paragraph on the available rabies control strategies to the introduction section:

- As dogs serve as the main source of human rabies, preventing and controlling canine rabies will inevitably prevent human rabies. High-coverage dog vaccination has been widely recommended to eliminate canine rabies. The World Health Organization (WHO) suggested that an effective vaccination program should reach at least 70% of the population [19]. In various contexts, however, different vaccination coverages, as well as vaccination strategies, may be required. Dog sterilization is an approach for controlling the reproduction of the dog population, specifically to stabilize or reduce the population size. In India, for example, using this method in conjunction with dog vaccination proved beneficial in controlling rabies [20]. Alternatively, dog culling has been practiced, even though this approach was unsuccessful in several areas [16, 18, 21] In China, dog vaccination programs alone might be insufficient to control rabies; however, dog culling in combination with vaccination has been recommended to enhance the rabies control [22].

Comment 3-1: "*The authors state that because the dog population in these two districts is unknown, they derive from the distribution of buildings how many owned and stray dogs are located in the study sites. This results in a dogs population of mainly owned dogs and very few stray dogs (compared to the owned dogs). The number of dogs in the study sites should be reported earlier than at line 314, ideally at the position where the number of buildings are given…*"

Response: Revised as suggested. The estimated number of dogs has now been added at the bottom of the first paragraph of the section "Geographical characteristics of Hatyai and Tepha districts," where the number of buildings in each group was provided.

- … there are 152,439 buildings in Hatyai. Among these 152,439 buildings, 144,532 (94.81%) are group-1 (G1) buildings (e.g., houses, residences, and other small buildings), 887 (0.58 %) are group-2 (G2) buildings (e.g., schools, temples, markets), and 7,020 (4.61 %) are group-3 (G3) buildings (e.g., groceries, hotels, banks) (Fig 2b). The Tepha district covers an area of 978 km^2. Although Tepha has a larger geographical area than Hatyai, it comprises only 33,652 buildings (Fig 2c). Of these, 33,275 (98.88 %) are G1 buildings, 75 (0.22 %) are G2 buildings, and 302 (0.90 %) are G3 buildings. In addition, based on the number of buildings, the estimated number of dogs in Hatyai was 207,818 and it was 48,142 in Tepha.

Comment 3-2: *"… A rough calculation shows that in Hatyai there are 24 times more owned that stray dogs, and in Tepha there are 68 times more owned dogs. Is this a realistic scenario? …"*

Response: According to a preliminary survey available from http://164.115.40.46/petregister, among 2,173,999 dogs surveyed, with owned 94.98 percent of the dogs are owned and only 5.02 percent are stray (19 times). The proportions of owned and stray dogs, on the other hand, vary by province. In Songkla , for example, the fraction is 6.25 times, while in Surin, Roi ed, and Chiang rai provinces, the fractions are 15.93, 48.83, and 67.03 times, respectively. In response to the reviewer's question, we believe that the 24 and 68 times are both possible.

Comment 3-3: "… *Also, the distribution of the dog population is random, considering the density of the different types of buildings (e.g., always 54% of the G1 buildings are occupied by dogs). Is this assumption also realistic? Aren't there clusters of dogs in the study site, where this proportion may be larger or lower? The same for the distribution of stray dogs.*"

Response: To our knowledge, we believe that there is no study about the spatial distribution of dogs in Thailand. For stray dogs, they typically live together around a public place. We therefore used the average number of dogs per public place of 10.

Comment 4-1: *"Distribution and behavior of stray dogs: It is assumed that the stray dogs are located at a given building and that they stay there (as pet dogs stay at their houses). Is this realistic? ..."*

Response: Yes, this is realistic in Thailand. We would like to provide more information that stray dogs in Thailand typically do not leave their territory to relocate to a new location, especially if they discover a location that provides them with food and a safe place to sleep. Dogs resting in the shade of temple trees or in front of a convenience store (with air conditioners) are typical sights in Thailand. Besides, the social behavior of the dogs allows them to stay in groups with some certain territories.

Comment 4-2: "*...Also, do stray dogs roam similarly than owned dogs? I would have expected that they roam further.*"

Response: Yes, we believe that free-roaming owned dogs have the ability to go as far as stray dogs. Many dog owners leave their dogs unleashed and allow them to roam freely.

Comment 5-1: *"The large majority of model parameters are based on assumptions. I was wondering whether there are any possibilities from the literature on which the authors could base their assumptions."*

Response: We have now added references of some model parameters in Table 1. Table 1 has been revised, which now reads

Table 2. Parameters and the baseline values used in the rabies transmission model.

Parameters	Values	Reference
Natural birth rate (b)	2.28×10^{-4} day^{-1}	[1]
Mortality rate (m)	$m = bS/N$	-
Importation rate (ε)	0.0082 day^{-1}	estimated based on incidence data

Latency rate (*σ*)	0.0448 day^{-1}	[2]
Rabies induced death rate (*δ*)	0.32 day^{-1}	[2]
Vaccination coverage	50.38 %	survey report (unpublished)
Transmission probability (*p*)	1×10^{-5}	model fitting
Mean travelling distance of a susceptible dog		
- living in a building near roads with confinable fences	0.1 km	assumption
- living in a building near roads without confinable fences	1 km	assumption
- living in a building far from roads with confinable fences	0.05 km	assumption
- living in a building far from roads without confinable fences	0.5 km	assumption
Mean travelling distance of a rabid dog		
- living in a building near roads with confinable fences	0.1 km	assumption
- living in a building near roads without confinable fences	5 km	assumption
- living in a building far from roads with confinable fences	0.05 km	assumption
- living in a building far from roads without confinable fences	2.5 km	assumption
Proportion of household with fence	0.48	[3]
Proportion of G1 buildings that own dogs	0.54	[4]
Proportion of G2 buildings that own dogs	0.9	assumption
Proportion of G3 buildings that own dogs	0.1	assumption
Average number of dogs per dog-owning household	2.67	[3]
Average number of dogs per public place	10	assumption
Average number of dogs per G3 building	1	assumption

For the importation rate, we used the phrase " estimated based on incidence data " instead of "assumption" since the importation rate was estimated using rabies incidence data obtained from the Department of Livestock Development of Thailand. We defined an imported case as the case that occurred after a prior case longer than 26 days (22.3 days of the incubation period [2] plus 3.1 days of the infectious period [2]).

For the proportion of households with fences and the average number of dogs per dog-owning household, the values of these two parameters were acquired from the field observations in Ref. [3]. At the time of this manuscript submission, this reference was not available; however, it has now been published, and the reference for values of these parameters has been added.

In addition, the sensitivity analysis has been performed for the model parameters that empirical data are not available. These parameters include the mean traveling distances of dogs, the proportion of buildings that own stray dogs, and the average number of stray dogs per building owning stray dogs. The results of the sensitivity analysis are shown in Figures S1 – S7 in the supplementary information. The unpublished survey report of vaccination coverage is also provided as an attachment.

Comment 5-2: "*… There are a range of articles on the roaming behavior of free-roaming dogs from which the authors may take information on the distance that dogs travel. ...*"

Response: Despite the fact that there are several publications on roaming behavior of free-roaming dogs, the range of movement varies depending on the geographical environment. However, we have now revised our manuscript to include a sensitivity analysis for these factors (Fig S1 and S2 in the supplementary information). The following sentences have been added at the end of paragraph that we described the effect of the geographical distribution of buildings and roads on the rabies transmission dynamics:

- … As the exact traveling distances of dogs are not known, we also perform the sensitivity analysis of the traveling distance. We found that when the distances traveled by dogs increase, the numbers of cumulative cases and the likelihood of making a secondary infection also increase, and vice versa (see Fig S1Error! Reference source not found. in the supplementary information).

In the discussion section, the following paragraph below has been added.

- However, the main weakness of our study is the absence of empirical data for some model parameters, especially the mean traveling distances of dogs. The roaming ranges of dogs vary greatly depending on the surroundings. For example, the median distance from the residence of free-roaming domestic dogs in a rural area of northern Australia was reported at 63 meters [5] while, in Kenyan rangeland, the values were 309 meters on commercial ranches [6]. Using high-resolution proximity logger to track contact networks of free-ranging domestic dogs in two rural settings in Chad, the total ranges were found to be 20.77 km^2 for Kakale and 4.56 km^2 for Magrao [7], corresponding to circular areas of radius 2.57 km and 4.56 km, respectively. We therefore performed a sensitivity analysis on the dog traveling distances by scaling the mean traveling distances by factors 0.5 and 2. Our simulations demonstrated that longer travel lengths expand the distance that the disease may spread and result in higher numbers of cumulative cases and more dispersed distributions of cumulative rabies cases (Fig S1 and S2).

Comment 5-3: "*… Also, it looks strange to me that the average number of dogs per dog-owning household is an assumption (2.67). How did the authors assume that number?...*"

Response: The average number of dogs per dog-owning household as well as proportion of household with fence were acquired from field observations in [3]. As of the time of this manuscript's submission, reference [2] had not yet been published. But now the paper is published, and the reference has been added to Table 1.

Comment 5-4: "*… Also the birth rate is very specific, how was that value assumed? ...*"

Response: The reference for the birth has now been added in Table 1.

Comment 5-5: "*… For the transmission probability is it said "estimation". How did the authors estimate this value? Also, it looks to me very low, as the probability that rabies is transmitted through a bite of a rabid animal is 0.49 according to ref [39]. …*"

Response: We fitted our model to reported data to get the transmission rate. In Table 1, we use the word "model fitting" in place of "estimation" to make this point clearer.

According to [2] (the mentioned ref [39]), the probability mean a probability that an unvaccinated dog developed rabies after being bitten by an infectious animal while in this study, the transmission probability means a transmission probability given an encountering between a susceptible dog and a rabid dog. In general, the probability of disease transmission from an encounter is lower than the probability of disease transmission from a bite, since an encounter event has a chance of causing a bite, whereas a bite has a chance of causing disease transmission.

Comment 5-6: "*… Finally, I highly recommend to do a sensitivity analysis for these parameters to understand how the model reacts towards the change of the parameter values.*"

Response: Thanks the referee again for the advice. We have now performed sensitivity analysis as suggested. Figs S1-S7 in the supplementary information show a sensitivity analysis of model parameters, including mean dog travel distances, the proportion of buildings containing stray dogs (G2 and G3 buildings), and the average number of stray dogs per building with stray dogs.

Comment 6: "*One of the main findings is, according to the authors, that the dog density is essential for rabies spread. However, they also found that the reducing the dog population density does not seem to greatly influence the probability of making a secondary transmission after importation of a rabid dog. In addition, it is propagated in the literature that rabies is not density dependent. Finally, it was not clear to me whether the population reduction only considers owned or owned and stray dogs. Is the reduction been done randomly? I suggest that the authors more carefully discuss the (notably very interesting) finding of the dog density for rabies spread.*"

Response: We apologize if the information provided was confusing. In the way we reduced the population, the dogs were randomly removed, without regard to whether they were stray or owned. As suggested, the discussion on reducing dog density and other intervention strategies has been revised, which now reads

- In this study, we investigated three intervention strategies, i.e., dog population reduction, dog movement restriction, and mass vaccination. Despite dog density matters for rabies persistence, our results highlighted that reducing the dog population density could diminish the likelihood of rabies transmission. However, if the reduction proportion is inadequately high to clear susceptible dogs within the traveling length of rabid dogs, transmission is still feasible. For example, in Hatyai, where dog density is 243 dogs/km^2, culling half of the population leaves 381 dogs within a one-kilometer roaming radius of a rabid dog. Therefore, it indicated that rabies might not be able to be eliminated by only reducing the dog population. Our finding is in line with a previous study in rural Tanzania where the dog population density is as low as 1.36 dogs per km^2, which is 99% and 97% lower than the dog populations in Hatyai and Tepha district, respectively, canine rabies still circulates in this area [2]. This indicated that implementing only culling strategies might not be effective enough to eliminate rabies [2, 15-18].

Comment 7-1: *"Dog confinement: as for dog population reduction, I also find this aspect worthwhile to be investigated. But I have some questions: Why are confined dogs still able to roam 100m on average? This still allows them to contact other dogs Is this assumption correct?..."*

Response: In Thailand, it is typical that confined dogs are not totally contained. Some owners let their dogs unleashed and allowing them to roam outdoors. To this end, we also investigated the impact of the confining distances on the likelihood of making a secondary infection of an imported infected dog. The result is shown in Figures S1, S2, and S7 in the supplementary information.

Comment 7-2: *"…Is it also a realistic assumption that almost 50% of the owned dogs are confined? This again raise the issue that a sensitivity analysis of the model is very important to see the influence of the parameters on the model outcomes….*"

Response: The evidence that 48% of dog owners kept their pets in residence with a confinable fence was obtained from a survey conducted in [3]. However, it's worth investigating the influence of confinement fraction on transmission risk. We, therefore, have performed sensitivity analysis as suggested. The result is shown in Figure S7 in the supplementary information.

Comment 7-3: *"…Finally, it is not that surprising that 100% confinement of the dogs allow to eliminate a rabies outbreak. However, it should be more discussed that even though the confinement is not 100% (because they can still roam 100m on average) and even though stray dogs are around and not confined (if I understood this correctly), there is still a promising effect of the rabies control by this strategy. This, together with the practicability in Thailand to undertake confinement of all owned dogs, has to be better discussed."*

Response: Revised as suggested. The paragraph has been added to the discussion section.

- Owned dog confinement also has the potential to decrease the transmission likelihood. Owing to the dense aggregation area in Hatyai, limiting traveling distances of the owned dogs could cut more contacts among dogs than Tepha. We found that restricting owned dog movement could effectively mitigate the transmission in both areas. Moreover, rabies could be eliminated if all owned dogs are restricted inside their homes. Although in the context of Thailand, in which most houses have no fences, strict enforcement on confinement of owned dogs might be impracticable. Therefore, this strategy might need to be implemented in combination with other interventions.

Comment 8: "Only one type of road is considered in the model. I would expect that the size of road would make a difference. There may be roads that helps the dogs to roam (as considered in the here described model), but there may also be roads that are clear barriers for roaming (as e.g. shown in Laager et al (doi: 10.1371/journal.pntd.0006680)). In addition, the direction of the road is important for the direction of the pronounced roaming. I do not think that this all needs to be implemented in the model (as this is a complex procedure), but it should at least be discussed.

Response: To address this point, we have analyzed the characteristics of roads in our study areas. We found that there are three types of roads in Hatyai and Tepha, namely,

4) Primary highways which are part of the network connecting regions, provinces, districts, and other important destinations
5) Motorways or special highways which are high-capacity highways designed for high-speed traffic
6) Rural and Local highways, which are small local roads.

Alignments of each road type are shown below (and in Figure S8 in the supplementary information). There are 25.19 km of primary highways, 15.12 km of motorways, and 2,762.26 km of local roads in Hatyai, representing 0.9%, 0.54%, and 98.56%, respectively. In Tepha, the total length of motorways and local roads are 65.075 km (5.54%) and 1138.811 km (94.59%), respectively, and there are no primary roads in Tepha. As the majority of roads in these study sites are small local roads in which the traffic is not heavy, we think that the role of the road barrier may be small. To discuss about this point, the following sentences have been added to the last paragraph in the Discussion section:

- Like other modeling studies, there were some limitations in our study. Firstly, while roads show a role to facilitate dog movement [8-13], high-traffic roads, on the other hand, were pointed out to be major geographical obstacles that might disrupt dog roaming [14]. However, as the roads networks in our study sites were dominated by small local roads (98.56% for Hatyai and 94.59% for Tepha) (see Fig S8), we hence did not consider the possible barrier role of roads in our study.

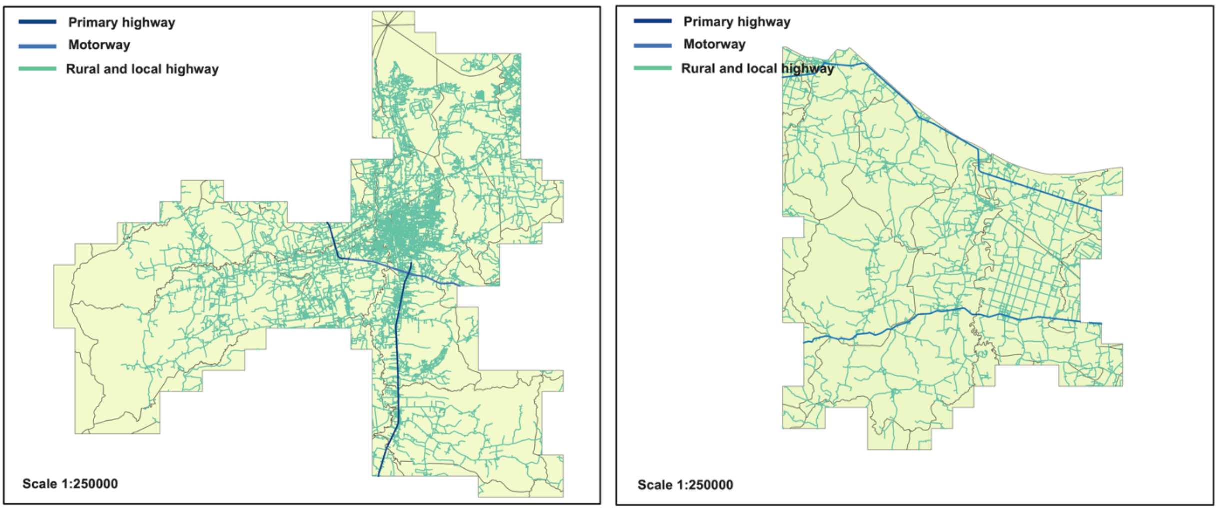

Fig. S8. Road alignments in (a) Hatyai and (b) Tepha. In Thailand, Roads are typically divided into 3 types, primary highways, motorways, and rural and local highways. Primary highways are part of the network connecting regions and provinces. Motorways are high-capacity highways designed for high-speed traffic. Rural and local highways are small local roads where traffics is usually not heavy. Therefore, dogs can usually cross and walk along the rural and local highways roads.

Comment 9-1: *"Writing style: There are some passages in all sections (introduction, M&M, results and discussion) that I would transfer to another section. E.g. line 108-121 better fits to the M&Ms; and at the end of the introduction it is more needed to state the aim of the study and its potential impact, rather than describing the model already here. ..."*

Response: Revised as suggested. The introduction section has been revised in accordance with the suggestions. Lines 108-121 have been removed since they are previously covered in the materials and methods section. The aim of the study has also been mentioned:

- In this study, we constructed a stochastic individual-based model to investigate the geo-temporal transmission dynamics of canine rabies in Thailand. The model integrates the exact geographical locations of buildings and alignments of roads. Two contrasting areas with high and low risk of rabies transmission in Thailand were chosen as case studies. In addition, we also investigated the impact of rabies interventions, including

dog vaccination, reducing the dog population, and restricting owned dog movement on the spread of canine rabies.

Comment 9-2: *"... Line 221-223 better fits to M&M. In general, the page 12 reads a bit as a mix of results and M&Ms."*

Response: In this subsection, we present the results of our analysis based on the GIS map that we received, such as the proportion of each category of buildings. As a result, we think that the content is more appropriate in this section. On the Line 221-223, We propose to restate a brief methodology before giving our findings.

Minor comments

Comment 1: "*Why did the authors took the probability of a secondary transmission as the outcome metric to investigate the effect of control actions, and not the number of rabies cases after a certain time of rabies propagation?*"

Response: Because the interventions we used in this study were static. Vaccination coverage, population size, and confinement proportion, for example, did not alter during the simulations. We want to investigate the potential of how an introduction of an infected dog can cause an outbreak in each setting.

Comment 2: "*The number of simulated cases in Hatyai indeed fits quite well with the reported data. It has been said that for Tepha it is not possible to undertake such a comparison, because no rabies cases are reported. However, it may also be argued that the number of cases is zero, and then it is possible to make a comparison with the conclusion that the model over-predicts the number of rabies cases. Also, what is the source of the number of cases in Hatyai.*

Response: As suggested by the reviewer, we have made comparison between simulation results and reported data in Tepha which was obtained from Department of Livestock Development (DLD).

- To investigate how different geographical distribution of buildings and roads could affect the spatiotemporal transmission dynamics of rabies, we separately simulated our rabies transmission model in Hatyai and Tepha using the same set of model parameters. We found that the number of locally transmitted rabid dogs in Hatyai simulated from our model generally agrees with the corresponding reported data (Fig 4 (a)). In Tepha, where rabies had been undetected for more than five years, the model prediction illustrated that there would be a very low chance for an outbreak in this district when an infected dog was imported (averaged cumulative local cases were fewer than one (Fig 4 (b)))…

Comment 3: "*Where are the randomly imported dogs located? Is this always an owned dog, or also stray? I think for both types of dogs an importation is possible.*"

Response: In this model, only stray dogs were imported to a random G2 or G3 building. The reviewer is correct, both types are possible.

Comment 4: "*When the term "significant" is used, please support it by a statistical test.*"

Response: Revised as suggested. We have removed the word "significant" when a statistical test cannot be provided.

Comment 5: "*Line 83-84: I suggest to edit: "... reservoir for rabies, rabies transmission to humans by dog bites may anytime happen, and the number…"* "

Response: Revised as suggested.

Comment 6: "*Line 92 (ref 15-18): only one of these references is related to dog rabies, all other are investigation wildlife rabies. This should be made clear, as wildlife are differently distributed than dogs, which depend on humans.*

Response: The sentence has been changed in accordance with the suggestion, which now reads

- In this regard, a spatial-dependent diffusion process was integrated into previous rabies transmission models both in wildlife populations [21-23] and dog populations [24].

Comment 7**:** "*Line 136: Please delete "where stray dogs usually can be found" because this comes later and raises question about the role of the other buildings.*"

Response: We appreciate your feedback. However, we have revised the following paragraph to make it clearer:

- The geographical maps aggregating layers of administrative boundaries, polygons representing buildings, and polyline of roads were retrieved from the Department of Public Works and Town & Country Planning, Ministry of Interior, Thailand. As we classified dogs into owned and stray, we thus categorized the buildings into three groups according to places where dogs usually reside. The first group (G1) contains houses and residential buildings. The second group (G2) involves public places where stray dogs can usually be found as there are ample food and shelters. This group of buildings comprises temples, schools, and fresh markets. Finally, the third group (G3) holds other types of buildings (e.g., groceries, hotels, banks, etc.)…

Comment 8**:** "*Line 148-149: please better describe the distributions of the stray dogs in the different buildings, as the number of dogs in G2 and G3 differs. This can only be found in table 1, thus it is important to refer to the table at this position.*"

Response: The "Population and spatial distribution of dogs" section has been revised, which now reads

- Our rabies transmission model considers both owned and stray dogs. Owned dogs refer to dogs with owners. They were assumed to be found only in G1 buildings. The number of owned dogs was calculated by multiplying the number of G1 buildings, the proportion of G1 buildings owning dogs, and the average number of owned dogs per household. Nevertheless, some of the dog owner's residences have no confinable fences allowing their pets to wander freely outside. A proportion of the G1 buildings were then

randomly chosen to be enclosed by fences, consequent in the shorter range of the dog movement (see Table 1).

- For stray dogs, although they were ownerless, they are usually fed by local feeders, e.g., Buddhist monks and kind aunties [23]. Similar to the owned dogs, the numbers of stray dogs were estimated based on the number of buildings, the proportion of buildings that own dogs, and the average number of stray dogs per dog-owning buildings (see Table 1). Based on our estimation, a majority (> 90%) of stray dogs were assumed to live in G2 buildings, while the rest live in G3 buildings (Fig 1(a)). The G2 and G3 buildings have no confinable fences, and all of the stray dogs are not contained.

Comment 9: "*Line 154: Please delete "in this work, "* "

Response: Revised as suggested.

Comment 10: "*Line 165: I suggest to use another symbol than d for the death rate, so that it cannot be mixed up with the distance d. Please describe that the death rate was selected as being the size to keep the population constant (I guess).*"

Response: Revised as suggested. We now use "mortality rate" and abbreviate it with "m." We also provide further information regarding the rate, which now read:

- … To maintain a constant size of the dog population, dogs in all classes were assumed to naturally die at a mortality rate $m = bS(t)/N(t)$.. …

Comment 11: "*Figure 1b: please include the death rate into the model scheme.*

Response: Revised as suggested.

Comment 12: "*Line 167: note that Kij is the encounter rate.*"

Response: Revised as suggested.

Comment 13: "*Line 210: I suggest to write "... the model classifies each single dog into susceptible, ...""*"

Response: Revised as suggested.

Comment 14: "*Line 237-238: The authors should explain what they mean with "different order of closeness".*"

Response: We now used word "rank" instead of "order". Figure 3(c) has also been revised to enhance the understanding, which now reads

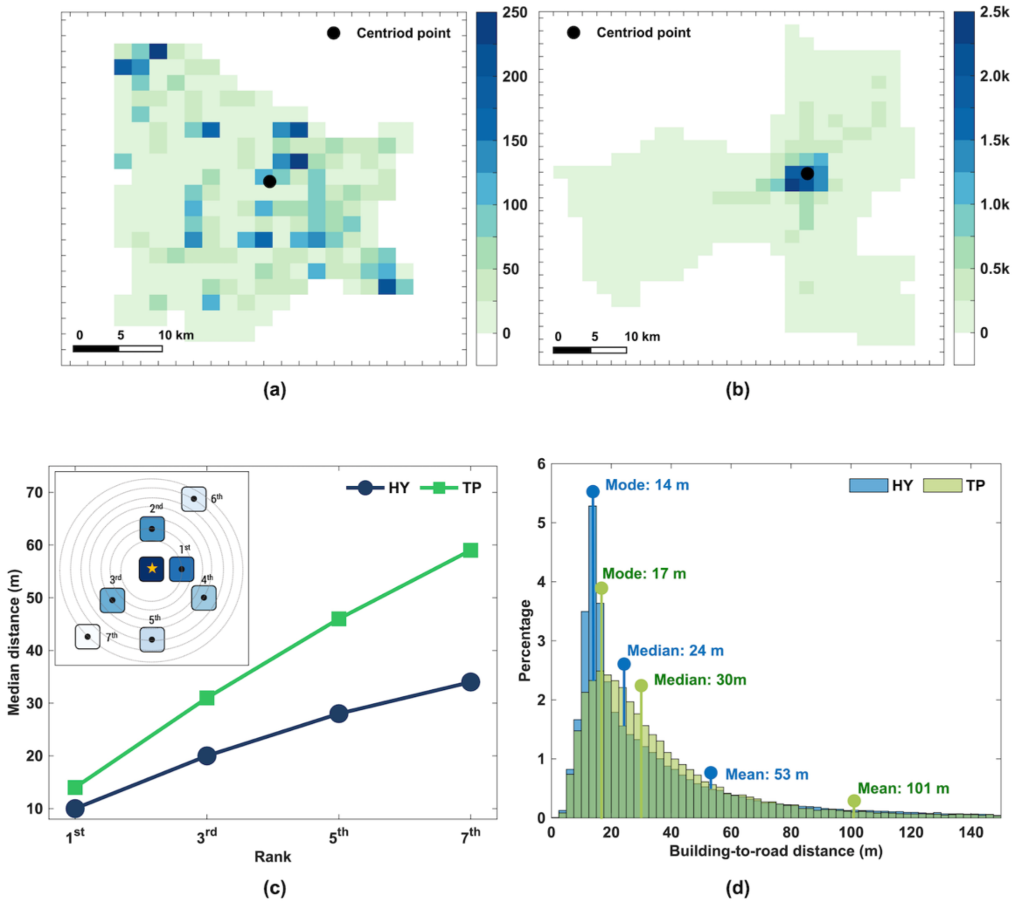

Fig 3. Building density and distribution. (a-b) Spatial distribution of building density. The color bar indicates the building density in the unit of buildings per square kilometer. The black circles show the centroid points of the building distribution. (c) The median distance between pairs of buildings with a different rank of closeness in Hatyai (HY) and Tepha (TP) district. The inset illustrates an example of a building arrangement with different ranks of closeness relative to the stared building. (d) The distribution of the shortest building-to-road distance in Hatyai (HY) and Tepha (TP).

Comment 15: "*Line 248-249: can be deleted, is a repetition.*"

Response: Revised as suggested.

Comment 16: "*Line 271-273: can be deleted (repetition)*"

Response: Revised as suggested.

Comment 17: "*Line 348: rabid instead of rapid*"

Response: Revised as suggested.

Comment 18: "*Line 348: these references only consider wildlife, not dog rabies*

Response: We apologize for the mistake; the sentence has now been corrected.

- We derived the encounter rate of a rabid dog and a susceptible dog that live at different locations based on the assumption of the random movement of rabid animals [24-26].

Comment 19: "*Lines 351-363: this is repetition and could be deleted* "

Response: We believe it is appropriate to restate the reasons for our site selections as well as our briefly findings here.

Comment 20: "*Line 369: please delete "typically"*

Response: Revised as suggested.

Comment 21: "*Line 370: put a comma after "dogs" and delete "so"* "

Response: Revised as suggested.

Comment 22: "*Line 401: it is said that the geographical setting matters for rabies spread. On the other hand, one may also argue that the conclusions from the study in regards to control measures tested are the same in both regions. Thus the difference in the geographical setting between the two study sites tested here does not result in different conclusions.*"

Response: Both locations have diverse geographical settings; however, the intervention has been randomly implemented regardless of the differences in geographical preference. That may be a reason why there is no obvious difference in the outcomes at the two sites. However, since our model has incorporated the geographical features, it can be a tool to explore the impact of control strategies regarding the geographical preference.

Comment 23: "*The limitation of not considering vaccination nor "immediate response" is mentioned here. But it is not discussed what may be the effect of this non-consideration. Also, I was wondering whether the author could calculate the average reproductive ratios from their model to have an idea how much this corresponds with what is expected.*"

Response. We have now included dog vaccination in our study. Since our model is a spatially explicit individual-based model, to our knowledge, we believe that we cannot calculate the average reproductive ratio (R) explicitly. In addition, since the number of rabid dogs is very low, this might result in a wide range of statistical uncertainty of R.

References

1. Michell, A.R., *Longevit of British breeds of dog and its relationships with-sex, size, cardiovascular variables and disease.* Veterinary Record, 1999. **145**(22): p. 625-629.
2. Hampson, K., et al., *Transmission Dynamics and Prospects for the Elimination of Canine Rabies.* PLOS Biology, 2009. **7**(3): p. e1000053.
3. S. Premashthira, S.S., W. Thanapongtharm, O. Sagaraseranee, W. Thichumpa, C. Sararat and A. Wiratsudakul, *The impact of socioeconomic factors on knowledge, attitudes, and practices of dog owners on dog rabies control in Thailand.* Front. Vet. Sci., 2021.
4. Kongkaew, W., et al., *Vaccination coverage and epidemiological parameters of the owned-dog population in Thungsong District, Thailand.* Prev Vet Med, 2004. **65**(1-2): p. 105-15.
5. Maher, E.K., M.P. Ward, and V.J. Brookes, *Investigation of the temporal roaming behaviour of free-roaming domestic dogs in Indigenous communities in northern Australia to inform rabies incursion preparedness.* Scientific Reports, 2019. **9**(1): p. 14893.
6. Woodroffe, R. and C.A. Donnelly, *Risk of contact between endangered African wild dogs Lycaon pictus and domestic dogs: opportunities for pathogen transmission.* Journal of Applied Ecology, 2011. **48**(6): p. 1345-1354.
7. Wilson-Aggarwal, J.K., et al., *High-resolution contact networks of free-ranging domestic dogs Canis familiaris and implications for transmission of infection.* PLOS Neglected Tropical Diseases, 2019. **13**(7): p. e0007565.
8. Brunker, K., et al., *Landscape attributes governing local transmission of an endemic zoonosis: Rabies virus in domestic dogs.* Mol Ecol, 2018. **27**(3): p. 773-788.
9. Brunker, K., et al., *Integrating the landscape epidemiology and genetics of RNA viruses: rabies in domestic dogs as a model.* Parasitology, 2012. **139**(14): p. 1899-1913.
10. Brunker, K., et al., *Landscape attributes governing local transmission of an endemic zoonosis: Rabies virus in domestic dogs.* Molecular ecology, 2018. **27**(3): p. 773-788.
11. Hassine, T.B., et al., *Rabies in Tunisia: A spatio-temporal analysis in the region of CapBon-Nabeul.* Acta Tropica, 2021. **216**: p. 105822.
12. Numminen, E. and A.-L. Laine, *The spread of a wild plant pathogen is driven by the road network.* PLOS Computational Biology, 2020. **16**(3): p. e1007703.
13. Fooks, A.R. and A.C. Jackson, *Rabies: Scientific Basis of the Disease and Its Management*. 2020: Elsevier Science & Technology.
14. Laager, M., et al., *A metapopulation model of dog rabies transmission in N'Djamena, Chad.* Journal of Theoretical Biology, 2019. **462**: p. 408-417.
15. Dürr, S. and M.P. Ward, *Development of a Novel Rabies Simulation Model for Application in a Non-endemic Environment.* PLOS Neglected Tropical Diseases, 2015. **9**(6): p. e0003876.
16. Morters, M.K., et al., *Evidence-based control of canine rabies: a critical review of population density reduction.* Journal of Animal Ecology, 2013. **82**(1): p. 6-14.
17. Townsend, S.E., et al., *Designing programs for eliminating canine rabies from islands: Bali, Indonesia as a case study.* PLoS Negl Trop Dis, 2013. **7**(8): p. e2372.
18. Zinsstag, J., et al., *Transmission dynamics and economics of rabies control in dogs and humans in an African city.* Proc Natl Acad Sci U S A, 2009. **106**(35): p. 14996-5001.
19. World Health, O., et al., *Global elimination of dog-mediated human rabies: report of the rabies global conference, 10-11 December 2015, Geneva, Switzerland*. 2016, World Health Organization: Geneva.
20. Totton, S.C., et al., *Stray dog population demographics in Jodhpur, India following a population control/rabies vaccination program.* Preventive Veterinary Medicine, 2010. **97**(1): p. 51-57.
21. Dalla Villa, P., et al., *Free-roaming dog control among OIE-member countries.* Preventive Veterinary Medicine, 2010. **97**(1): p. 58-63.
22. Hou, Q., Z. Jin, and S. Ruan, *Dynamics of rabies epidemics and the impact of control efforts in Guangdong Province, China.* Journal of Theoretical Biology, 2012. **300**: p. 39-47.
23. Komol, P., et al., *The spread of rabies among dogs in Pranburi district, Thailand: a metapopulation modeling approach.* Frontiers in veterinary science, 2020. **7**.
24. Fofana, A.M. and A. Hurford, *Mechanistic movement models to understand epidemic spread.* Philosophical Transactions of the Royal Society B: Biological Sciences, 2017. **372**(1719): p. 20160086.
25. Murray, J.D., E.A. Stanley, and D.L. Brown, *On the spatial spread of rabies among foxes.* Proceedings of the Royal Society of London. Series B. Biological Sciences, 1986. **229**(1255): p. 111-150.
26. Miller Neilan, R. and S. Lenhart, *Optimal vaccine distribution in a spatiotemporal epidemic model with an application to rabies and raccoons.* Journal of Mathematical Analysis and Applications, 2011. **378**(2): p. 603-619.
