# Supplementary material for: The effects of geographical distributions of buildings and roads on the spatiotemporal spread of canine rabies: An individual-based modeling study
Source: PLoS Negl Trop Dis. 2022 May 10;16(5):e0010397. doi: 10.1371/journal.pntd.0010397 (PMC9126089; doi:10.1371/journal.pntd.0010397)
Supplement: Attachment — Submitted filename: Response to the Comments2.docx [file pntd.0010397.s012.docx]

**Response to the Editors’ Comments**

**Editors’ comments**

“*Thank you for your revised manuscript which has addressed many of the reviewer's comments. Please address those that remain below.*”

**Response:** We thank the Editors for giving us the opportunity to submit a revised version of our work. We appreciate the time and effort that the Editors and the Reviewers dedicated to providing feedback on our manuscript. We have now revised the manuscript in view of the comments. Please see the responses to the comments below.

**Suggestion 1:** “*In particular, the issue of lack of empirical data supporting the assumptions remains. I suggest that an explanation for the assumptions around dog movement distances are clearly included in the methods, and parameter table 1 (supported where appropriate by empirical data from other studies referenced in lines 402-414, and reference 35 and local information to put them into regional context).*”

**Response:** As the Editor suggested, the assumptions about dog mobility have been now described in the method section. The following paragraph has been added to the Model structure subsection:

- Dog roaming behaviors might be different in various environments. Free-ranging domestic dogs in rural areas in Chad, for example, have home ranges that correspond to a few kilometers of a circular radius [1], whereas free-roaming domestic dogs in Chad, Guatemala, Indonesia, and Uganda move little more than one hundred meters from their home [2]. In the lack of empirical data of dog movement in our study sites, we assumed that free-roaming dogs, i.e., dogs residing in buildings without confinable fences, travel at a mean distance of 500 meters from their homes. Confined owned dogs, i.e., dogs living in the G1 buildings with confinable fences, were assumed to travel around their home territory with a mean distance of 50 meters. Our model assumed that roads would facilitate dog wandering [3-7]; thus, dogs living in buildings near roads could travel further than dogs far from roads. (**Table 1**). However, a sensitivity analysis of the dog's traveling distance was also performed by scaling the mean traveling distances by factors of 0.5 and 2. Note also that although there is a study pointed out that high-traffic roads could be major geographical obstacles to dog roaming [8], the road networks in our study areas are dominated by small local roads (96.65 % for Hatyai and 94.59 % for Tepha, Fig S1), we hence did not consider the possible barrier role of roads in our study.

Table 1 has also been revised to include the details and references or model parameters. A sensitivity analysis for a parameter whose value cannot be found from the literature is now clearly stated in Table 1. Table 1 now reads

**Table 1**. Parameters and the baseline values used in the rabies transmission model.

| **Parameters** | **Values** | **Details and references** |
| --- | --- | --- |
| Natural birth rate (*b*) | 2.28×10^-4^ day^-1^ | [9] |
| Mortality rate (*m*) | *m = bS/N* | - |
| Importation rate (*ε*) | 0.0082 day^-1^ | Estimated based on incidence data |
| Latency rate (*σ)* | 0.0448 day^-1^ | [10] |
| Rabies induced death rate (*δ)* | 0.32 day^-1^ | [10] |
| Vaccination coverage | 50.38 % | Survey report |
| Model parameter representing the likelihood that a susceptible dog will be infected by an infectious dog (*p*) | 1×10^-5^ | Model fitting with R-square = 0.9772 |
| Mean travelling distance of a susceptible dog |  | Assumed, with sensitivity analysis shown in Figures S2 and S3 |
| - living in a building near roads with confinable fences | 0.1 km |  |
| - living in a building near roads without confinable fences | 1 km |  |
| - living in a building far from roads with confinable fences | 0.05 km |  |
| - living in a building far from roads without confinable fences | 0.5 km |  |
| Mean travelling distance of a rabid dog |  | Assumed, with sensitivity analysis shown in Figures S2 and S3 |
| - living in a building near roads with confinable fences | 0.1 km |  |
| - living in a building near roads without confinable fences | 5 km |  |
| - living in a building far from roads with confinable fences | 0.05 km |  |
| - living in a building far from roads without confinable fences | 2.5 km |  |
| Proportion of household with fence | 0.48 | [11] |
| Proportion of G1 buildings that own dogs | 0.54 | [12] |
| Proportion of G2 buildings that own dogs | 0.9 | Assumed, with sensitivity analysis shown in Figure S4 |
| Proportion of G3 buildings that own dogs | 0.1 | Assumed, with sensitivity analysis shown in Figure S5 |
| Average number of dogs per dog-owning household | 2.67 | [11] |
| Average number of dogs per G2 building | 10 | Assumed, with sensitivity analysis shown in Figure S6 |
| Average number of dogs per G3 building | 1 | Assumed, with sensitivity analysis shown in Figure S7 |

To avoid repetition of content, Lines 402-409 of the fifth paragraph of the discussion section have been removed. The messages on lines 410-414 were moved to the end of the fourth paragraph.

**Suggestion 2:** “*The sensitivity analyses are a worthwhile addition demonstrating a large effect on transmission. The reader will be interested in their effect on the main conclusions (Figure 6). If uncertainty over dog movements has a significant impact on the relative success of interventions, then that is an important conclusion in itself.*”

**Response:** The sensitivity analyses of dog traveling distances on the effectiveness of interventions have now been included in Figure 6. Figure 6 now reads

**Fig 6. Impact of intervention strategies on the likelihood of rabies transmission in Hatyai (a-c) and Tepha (d-f).** The likelihood of rabies transmission caused by an imported infected dog under the intervention scenarios of (a, d) reducing the dog population size, (b, e) reducing the fraction of free-roaming dogs, and (c, f) increasing the mass vaccination coverage. A sensitivity analysis where the dog traveling distances are scaled by factors of 0.5 (x0.5) and 2 (x2) was also shown (dashed lines).

To discuss this new sensitivity analysis result, the following sentences have been added to the first, second, and third paragraphs in the subsection of “Effect of canine rabies interventions”, respectively:

- The same tendency was also found for different dog-traveling ranges; however, the longer dogs can go, the greater the chance of transmission.
- We also found that the traveling ranges of dogs have greater influences on the transmission risk when the confinement fraction is small.
- Consistently, when more dogs are protected by the vaccine, the distances dogs can travel less affects the probability of transmission. However, shorter dog-travel distances help reduce the likelihood of disease transmission in places where vaccination coverage is low.

**Suggestion 3:** “*I also recommend using 'free-roaming' rather than 'stray'.*”

**Response:** We agree with the Editor that the word “stray” is inappropriate in our context. To address this point and make it more straightforward for the readers, we have now clearly described the classification and characteristics of dogs in our study. The subsection “Population and spatial distribution of dogs” has been thoroughly revised, which now reads

- In our model, dogs are classified into three types: owned dogs, owned free-roaming dogs, and unowned free-roaming dogs. Owned dogs were assumed to be found in G1 buildings that have confinable fences, whereas owned free-roaming dogs could be found in G1 buildings with no confinable fences. The number of owned dogs was calculated by multiplying the number of G1 buildings with fences, the proportion of G1 buildings owning dogs, and the average number of owned dogs per household. Similarly, the number of owned free-roaming dogs was estimated by multiplying the number of G1 buildings with no fences, the proportion of G1 buildings owning dogs, and the average number of owned dogs per household.
- For unowned free-roaming dogs, although they were ownerless, they are usually fed by local feeders, e.g., Buddhist monks and kind aunties [13]. Therefore, these dogs live near public places (G2 or G3 buildings) where local feeders usually provide them food. Since G2 and G3 buildings have no confinable fences, these unowned dogs can roam freely. The numbers of unowned free-roaming dogs residing in G2 and G3 buildings were estimated based on the number of buildings in each category, the proportion of buildings that own dogs, and the average number of unowned free-roaming dogs per dog-owning buildings (Table 1). Based on our assumption, about 91% of unowned free-roaming dogs in Hatyai and 99% of unowned free-roaming dogs in Tepha live in G2 buildings, while the rest live in G3 buildings (Fig 1 (a)).

The word “stray” has been changed to “unowned free-roaming” throughout the manuscript.

**Response to the Reviewers’ Comments**

**Reviewer #1**

**Methods**

**Comment 1**: *“The authors have made a massive effort in revising this paper. However, I have still a major reservation because of the large number of assumptions the authors still make. I recommend the authors to collect more primary data to substantiate their assumptions. In my own work my assumptions were often wrong and we must first observe nature and understand it deeply prior to attempting to model it.*”

**Response:** Although we have made several assumptions on our model parameters, the sensitivity analyses have been performed for most of the unknown parameters in this revised version of our manuscript. Sensitivity analysis is usually employed to find the relation between the uncertainty in a model-based outcome and the uncertainties in the model assumptions, especially in epidemic modeling [14]. To make it clear for the readers about our parameter values and the sensitivity analyses, Table 1 has been revised to include details in sensitivity analyses, which now reads

**Table 1**. Parameters and the baseline values used in the rabies transmission model.

| **Parameters** | **Values** | **Details and references** |
| --- | --- | --- |
| Natural birth rate (*b*) | 2.28×10^-4^ day^-1^ | [9] |
| Mortality rate (*m*) | *m = bS/N* | - |
| Importation rate (*ε*) | 0.0082 day^-1^ | Estimated based on incidence data |
| Latency rate (*σ)* | 0.0448 day^-1^ | [10] |
| Rabies induced death rate (*δ)* | 0.32 day^-1^ | [10] |
| Vaccination coverage | 50.38 % | Survey report |
| Model parameter representing the likelihood that a susceptible dog will be infected by an infectious dog (*p*) | 1×10^-5^ | Model fitting with R-square = 0.9772 |
| Mean travelling distance of a susceptible dog |  | Assumed, with sensitivity analysis shown in Figures S2 and S3 |
| - living in a building near roads with confinable fences | 0.1 km |  |
| - living in a building near roads without confinable fences | 1 km |  |
| - living in a building far from roads with confinable fences | 0.05 km |  |
| - living in a building far from roads without confinable fences | 0.5 km |  |
| Mean travelling distance of a rabid dog |  | Assumed, with sensitivity analysis shown in Figures S2 and S3 |
| - living in a building near roads with confinable fences | 0.1 km |  |
| - living in a building near roads without confinable fences | 5 km |  |
| - living in a building far from roads with confinable fences | 0.05 km |  |
| - living in a building far from roads without confinable fences | 2.5 km |  |
| Proportion of household with fence | 0.48 | [11] |
| Proportion of G1 buildings that own dogs | 0.54 | [12] |
| Proportion of G2 buildings that own dogs | 0.9 | Assumed, with sensitivity analysis shown in Figure S4 |
| Proportion of G3 buildings that own dogs | 0.1 | Assumed, with sensitivity analysis shown in Figure S5 |
| Average number of dogs per dog-owning household | 2.67 | [11] |
| Average number of dogs per G2 building | 10 | Assumed, with sensitivity analysis shown in Figure S6 |
| Average number of dogs per G3 building | 1 | Assumed, with sensitivity analysis shown in Figure S7 |

We believe that our employed modeling methodology, together with these sensitivity analyses, should be technically sound. However, to make it clear from the first glance that our work is a modeling study, the manuscript title has been revised, which now reads

- The effects of geographical distributions of buildings and roads on the spatiotemporal spread of canine rabies: An individual-based modeling study

**Comment 2**: “*The authors maintain an importation parameter in their model that reflects the human mediated dog transport. The claim that this is estimated based on incidence data. But how can they distinguish between the incidence provided from local chains of transmission and the importation from the outside? It would make a lot of sense in this model to collect empirical dog importation data first.*”

**Response:** We would like to explain, in our study, a rabid dog that was identified after 25.4 days (i.e., sum of the latent period and infectious period) since the previous most recent reported rapid dog was assumed to be an imported case. We think that the chance that two successive local transmitted cases are separated longer than 25.4 days should be unlikely.

The following sentences have been added at the end of the first paragraph of the subsection of the Model structure to clarify this point:

- The importation rate was estimated from the incident data where a rabid dog that was identified after 25.4 days (i.e., sum of the latent period and infectious period) since the previous most recent reported rapid dog was assumed to be an imported case. All imported cases were assumed to be unowned free-roaming dogs.

**Comment 3**: “*The authors explain the different types of roads in the study area, but we still don't know which of these roads are facilitating dog movement and which ones are barriers for dog movement. Again more empirical data on this would make the models much more realistic.*”

**Response:** This comment is similar to Comment 8-1 and Comment 8-2 of Reviewer #2. We would like to more provide more information that roads in Thailand are categorized and labeled with number digit(s). Routes connecting Bangkok to outlying regions are denoted with a single-digit highway, such as route 4 to the south. A two-digit highway denotes a primary highway within a region. A three-digit highway is a regional secondary route, whereas a four-digit highway connects a provincial capital to its districts or to major places within the province. Finally, a local road is a roadway that is managed by a local administrative organization.

To see how different types of roads look like, we would like to provide samples of each kind of road using street views of Google map, as follows

- Single-digit highways or interregional highways
- Highway number 4 in Hatyai district: <https://goo.gl/maps/SXY54uXNssvqSVfS9>
- For Tepha district, there is no one-digit highway
- Two-digit highways or regional primary highways
- Route 43 in Hatyai district: <https://goo.gl/maps/61i3rMy4ZTdTpcov6>
- Route 42 in Tepha district: <https://goo.gl/maps/S9cHexM1ASPwMqWW9>
- Route 43 in Tepha district: <https://goo.gl/maps/rbZAs1m53FK9oGuq5>
- Three-digit highways or regional secondary highways
- Route 407 in Hatyai district: <https://goo.gl/maps/AbvFsq45Ehy5mq5y8>
- Four-digit local roads (intra-province roads)
- Route 4046 in Hatyai district: <https://goo.gl/maps/NfHTV9VCEaZdT5A66>
- Route 4085 in Tepha district: <https://goo.gl/maps/GYxC1mUaHXxvGDCD7>
- Local road that organized by the local administrative organization.
- Local road in Hatyai district: <https://goo.gl/maps/qpq4jHbmRTR56ie26>
- Local road in Tepha district: <https://goo.gl/maps/4ZFjHDKLuL5ueDqJ8>

Because local roads are often narrow and the traffic is very light, we do believe they serve as facilitators rather than obstacles for dog movement. Traffic in our study sites is low to modest even at rush hour. So, dogs may also cross or walk along the kind of roads.

To discuss this point, the following changes have been made:

The following sentences have been added to the 3^rd^ paragraph in the subsection of “Model structure”:

- Our model assumed that roads would facilitate dog wandering [3-7]; thus, dogs living in buildings near roads could travel further than dogs far from roads. (**Table 1**). However, a sensitivity analysis of the dog's traveling distance was also performed by scaling the mean traveling distances by factors of 0.5 and 2. Note also that although there is a study pointed out that high-traffic roads could be major geographical obstacles to dog roaming [8], the road networks in our study areas are dominated by small local roads (96.65 % for Hatyai and 94.59 % for Tepha, Fig S1), we hence did not consider the possible barrier role of roads in our study.

The following sentences have been added to the last paragraph in the Discussion section:

- Like other studies, there were some limitations in our study. Firstly, we only consider the facilitator function of roads. Although our study sites are dominated by small local roads, some high-traffic roads could be geographical obstacles to dog roaming [8].

**Results**

**Comment 1**: “*I still feel at unease with this paper because the results still build on a large number of assumptions of which we don't know if they hold true. Again, I recommend the authors to collect first more empirical data on dog ecology in Thailand prior to develop a mathematical model.*”

**Response:** This comment is similar to Comment 1 in Methods of Reviewer #1. We would like to emphasize that although several assumptions on our model parameters have been made, the sensitivity analyses have now been performed for most of the model parameters. We believe that our employed modeling methodology, together with these sensitivity analyses, should be technically sound and appropriate.

In addition, the manuscript title has been revised to make it straightforward for the readers that our study focuses on rabies transmission modeling, which now reads

- The effects of geographical distributions of buildings and roads on the spatiotemporal spread of canine rabies: An individual-based modeling study

**Conclusions**

**Comment 1**: “*The authors have modified the conclusion by addressing dog mass vaccination as most important recommended strategy. This is however not a primary conclusion of this paper and hence the reader does not understand what is the ultimate purpose of this model. Although I recognize the hard work of revising this paper, I still feel that the knowledge gain is too small because of the high uncertainty with all the assumptions.*”

**Response:** To address this point, several changes have been made as follows.

We have revised the last paragraph in the introduction section to describe the study's aims clearly, which now reads

- In this study, we constructed a stochastic individual-based model that integrates the exact geographical locations of buildings and alignments of roads. The spatial distribution of dogs in the model was estimated according to the distribution of buildings, and roads were assumed to facilitate dog movement. The constructed model was then employed to investigate the geo-temporal transmission dynamics of canine rabies in two contrasting areas with high and low risk of rabies transmission in Thailand. Finally, the influence of rabies interventions such as dog vaccination, dog population reduction, and owned dog movement restrictions on the spread of canine rabies was explored.

Accordingly, the Author summary section has also been revised, which now reads

- … Our modeling results highlighted that only differences in the geographical structures of buildings and roads could result in the difference in rabies transmission dynamics in these two areas. We also explored the impacts of reducing dog population, restriction of owned dog movement, and dog vaccination on the rabies spread. We discovered that lowering the dog population alone might not be enough to keep rabies from spreading in the high-risk area. Owned dog confinement may lower the risk of rabies transmission even more. In addition, different levels of vaccination coverages are probably required to control rabies in different geographical settings.

The following sentence has been added at the end of the abstract:

- Finally, a higher vaccination coverage may be required for controlling rabies transmission in the high-risk area compared to the low-risk area.

The following sentence has been added to the second paragraph in the subsection of Effect of canine rabies interventions:

- We also found that the traveling ranges of dogs have greater influences on the transmission risk when the confinement fraction is small.

The following sentence has been added to the third paragraph in the subsection of Effect of canine rabies interventions.

- Consistently, when more dogs are protected by the vaccine, the distances dogs can travel less affects the probability of transmission. However, shorter dog-travel distances help reduce the likelihood of disease transmission in places where vaccination coverage is low.

The following sentence has been added to the second paragraph from the end of the Discussion section:

- Since the difference in the geographical distribution of buildings and roads could affect the transmission dynamics of rabies, planning control measures might need to account for this effect. In densely populated areas, for example, a higher level of implementation may be required.

**Reviewer #2**

The reviewer stated that “*Thank you for the great revision of the manuscript. Most of the concerns and uncertainties were cleared, however there are still a range of comments and open questions:* ”

**Comment 1**: “*I think it needs to be made clear that the link between geographical situation and rabies spread goes via the dog population simulated in the model. There the crucial assumption taken in the model that a high building and road density leads to a larger number of dogs and to more roaming, respectively. This needs to be indicated clearly from the start (also in the abstract). This is particularly important because the interventions are taken on the dogs, not on the buildings and roads. So it needs to be understood that dog population is explicitly modeled here.*”

**Response:** As suggested by the reviewer, the following sentences have been added to the abstract, author summary, and the last paragraph of the introduction:

- **Abstract**

In our model, the spatial distribution of dogs was estimated based on the distribution of buildings, with roads considered to facilitate dog movement.

- **Author summary**

The spatial distribution of dogs was assumed according to the distribution of buildings, with roads included to help dogs travel around.

- **Introduction**

In this study, we constructed a stochastic individual-based model that integrates the exact geographical locations of buildings and alignments of roads. The spatial distribution of dogs in the model was estimated according to the distribution of buildings, and roads were assumed to facilitate dog movement.

**Comment 2**: “*Line 36: was vaccination also only done in owned dogs?*”

**Response:** Actually, vaccinations were randomly performed in both owned and unowned dogs. To clarify this point, the following changes have been made:

The sentence in line 193 in the 1^st^ paragraph of the subsection of “Model structure” has been revised, which now reads

- Susceptible dogs, both owned and unowned, are vaccinated at a rate *ν*, while vaccinated dogs lost their vaccination immunity at a rate *ω*.

The second sentence in the 3^rd^ paragraph in the subsection of **“**Effect of canine rabies interventions” has been revised, which now reads

- As demonstrated in Fig 6 (c), when both owned and unowned dog populations were randomly vaccinated, the likelihood of an imported infected dog causing a secondary infection was lower with the increase of vaccination coverage and eventually disappeared when the whole population was immunized.

**Comment 3**: “*Line 37: Please explain how can these results direct policy.”*

**Response:** We thank the reviewer for pointing out this point. We think that this sentence might not be appropriate for the abstract. Therefore, the sentence “These findings are beneficial for the policy improvement to prevent and control canine rabies in Thailand and other countries with similar settings” has been replaced by “Finally, a higher vaccination coverage may be required for controlling rabies transmission in the high-risk area compared to the low-risk area.”

**Comment 4**: “*Line 94: Did this increase in the number of rabies cases happen without any change in the surveillance system?”*

**Response:** No, it was not from the changes in the surveillance system. We believe that it was due to the change in vaccine management policy. Previously, the local administrative organizations (LAO) could buy and allocate rabies vaccines in their areas. However, in 2014, the Office of the Auditor General (OAG) did not allow the LAO to purchase vaccines, and the number of rabies cases had increased since then (see more details at: <https://www.hiso.or.th/hiso/picture/reportHealth/ThaiHealth2019/eng2019_20.pdf>). Nonetheless, as the number of rabies cases had unprecedentedly increased, the OAG, later on, decided to authorize the LAO to help with the supply of rabies vaccines in their areas (<https://thainews.prd.go.th/en/news/detail/WNSOC6103150010024> ). Then, the cases had declined.

Unfortunately, there is still no scientific evidence for this association so far. We hence cannot put the explanation above in our paper. We can only describe what happened.

**Comment 5**: “*Line 120: can "wild animals" be replaced by raccoons?”*

**Response:** Revised as suggested.

**Comment 6**: “*Line 123: "…and alignments of roads." Suggest to add here " and use them to simulate the dog population in these area." Or similar.”*

**Response:** Revised as suggested. The last paragraph of the Introduction section has been revised, which now reads

- In this study, we constructed a stochastic individual-based model that integrates the exact geographical locations of buildings and alignments of roads. The spatial distribution of dogs in the model was estimated according to the distribution of buildings, and roads were assumed to facilitate dog movement. The constructed model was then employed to investigate the geo-temporal transmission dynamics of canine rabies in two contrasting areas with high and low risk of rabies transmission in Thailand. Finally, the influence of rabies interventions such as dog vaccination, dog population reduction, and owned dog movement restrictions on the spread of canine rabies was explored.

**Comment 7**: “*Line 135: How is the reporting of rabies cases done? What type of surveillance does exist in this region? This may give an idea of underreporting.”*

**Response:** As described in Premashthira et al., 2021 (DOI: 10.3389/fvets.2021.699352), the national rabies surveillance program in Thailand consists of both active and passive systems. However, most of the samples were collected passively.

**Comment 8-1**: “*Line 137: It is great that you assessed the quality of roads, but it should be described somewhere in the methods (probably in this section).*”

**Response:** Revised as suggested. We now have clearly described the roles of roads in the Methods section. The following sentences have been added to the 3^rd^ paragraph in the subsection of “Model structure”:

- Our model assumed that roads would facilitate dog wandering [3-7]; thus, dogs living in buildings near roads could travel further than dogs far from roads. (**Table 1**). However, a sensitivity analysis of the dog's traveling distance was also performed by scaling the mean traveling distances by factors of 0.5 and 2. Note also that although there is a study pointed out that high-traffic roads could be major geographical obstacles to dog roaming [8], the road networks in our study areas are dominated by small local roads (96.65 % for Hatyai and 94.59 % for Tepha, Fig S1), we hence did not consider the possible barrier role of roads in our study.

Figure S1 describing the classification of roads in Hatyai and Tepha has also been revised, which reads

**Fig. S1. Road alignments in (a) Hatyai and (b) Tepha**. In Thailand, roads are typically divided into 4 types, interregional highways, regional primary highways, regional secondary highways, and rural and local roads. Interregional highways are highways connecting Bangkok to outlying regions (for example, Route 4 to southern Thailand). Regional highways are highways within a region. Local roads are roads connecting main roads to important locations. The local roads are usually small roads where traffics is usually not heavy. Therefore, dogs can usually cross and walk along the roads. In the Hatyai area, there is 25.19 km of interregional highways, 15.12 km of regional primary highways, 53.74 km of regional secondary highways, and 2,708.52 km of local roads, representing 0.9%, 0.5%, 1.9%, and 96.7%, respectively. In Tepha, there are no interregional highways; the total length of regional highways and local roads is 65.08 km (5.4%) and 1,138.81 km (94.6%), respectively. The base layer of the map was obtained from <https://data.humdata.org/dataset/thailand-administrative-boundaries>.

**Comment 8-2**: “*I am however not sure if the correct thresholds are taken. I agree that the max 5% of large roads can be ignored, but I guess that there is also variation in the here called "local roads" in terms of building barriers for dogs. Such assumption that can be better discussed.*”

**Response:** We would like to more provide more information that roads in Thailand are categorized and labeled with number digit(s). Routes connecting Bangkok to outlying regions are denoted with a single-digit highway, such as route 4 to the south. A two-digit highway denotes a primary highway within a region. A three-digit highway is a regional secondary route, whereas a four-digit highway connects a provincial capital to its districts or to major places within the province. Finally, a local road is a roadway that is managed by a local administrative organization.

To see how different types of roads look like, we would like to provide samples of each kind of road using street views of Google map, as follows

- Single-digit highways or interregional highways
- Highway number 4 in Hatyai district: <https://goo.gl/maps/SXY54uXNssvqSVfS9>
- For Tepha district, there is no one-digit highway
- Two-digit highways or regional primary highways
- Route 43 in Hatyai district: <https://goo.gl/maps/61i3rMy4ZTdTpcov6>
- Route 42 in Tepha district: <https://goo.gl/maps/S9cHexM1ASPwMqWW9>
- Route 43 in Tepha district: <https://goo.gl/maps/rbZAs1m53FK9oGuq5>
- Three-digit highways or regional secondary highways
- Route 407 in Hatyai district: <https://goo.gl/maps/AbvFsq45Ehy5mq5y8>
- Four-digit local roads (intra-province roads)
- Route 4046 in Hatyai district: <https://goo.gl/maps/NfHTV9VCEaZdT5A66>
- Route 4085 in Tepha district: <https://goo.gl/maps/GYxC1mUaHXxvGDCD7>
- Local road that organized by the local administrative organization.
- Local road in Hatyai district: <https://goo.gl/maps/qpq4jHbmRTR56ie26>
- Local road in Tepha district: <https://goo.gl/maps/4ZFjHDKLuL5ueDqJ8>

Because local roads are often narrow and the traffic is very light, we do believe they serve as facilitators rather than obstacles for dog movement. Traffic in our study sites is low to modest even at rush hour. So, dogs may also cross or walk along the kind of roads.

To discuss this point, the following changes have been made:

The following sentences have been added to the 3^rd^ paragraph in the subsection of “Model structure”:

- Our model assumed that roads would facilitate dog wandering [3-7]; thus, dogs living in buildings near roads could travel further than dogs far from roads. (**Table 1**). However, a sensitivity analysis of the dog's traveling distance was also performed by scaling the mean traveling distances by factors of 0.5 and 2. Note also that although there is a study pointed out that high-traffic roads could be major geographical obstacles to dog roaming [8], the road networks in our study areas are dominated by small local roads (96.65 % for Hatyai and 94.59 % for Tepha, Fig S1), we hence did not consider the possible barrier role of roads in our study.

The following sentences have been added to the last paragraph in the Discussion section:

- Like other studies, there were some limitations in our study. Firstly, we only consider the facilitator function of roads. Although our study sites are dominated by small local roads, some high-traffic roads could be geographical obstacles to dog roaming [8].

**Comment 9**: “*Line 141: How was this categorization of buildings done? Visually by looking at the maps, or otherwise?*”

**Response:** According to the shapefiles we received from the Department of Public Works and Town & Country Planning (DPT), the buildings have already been assigned a classification based on their usage purposes. We just regrouped the buildings into G1, G2, and G3 using QGIS software (version 3.16.10).

**Comment 10**: “*Line 145: Please also state for the G3 buildings how they host dogs.*”

**Response:** Revised as suggested. The following sentences have been added,

- Finally, the third group (G3) holds other types of buildings, including groceries, hotels, banks, etc. Some unowned free-roaming dogs live near these buildings as people sometimes feed them with some foods.

**Comment 11**: “*Line 162 (or close by): There is a sentence missing saying that a proportion of owned dogs are modelled to not roam outside (if this is correct).*”

**Response:** This comment is similar to suggestion 3 of the Editor. To address this point and make it more straightforward for the readers, we have now clearly described the classification and characteristics of dogs in our study. The subsection “Population and spatial distribution of dogs” has been thoroughly revised, which now reads

- In our model, dogs are classified into three types: owned dogs, owned free-roaming dogs, and unowned free-roaming dogs. Owned dogs were assumed to be found in G1 buildings that have confinable fences, whereas owned free-roaming dogs could be found in G1 buildings with no confinable fences. The number of owned dogs was calculated by multiplying the number of G1 buildings with fences, the proportion of G1 buildings owning dogs, and the average number of owned dogs per household. Similarly, the number of owned free-roaming dogs was estimated by multiplying the number of G1 buildings with no fences, the proportion of G1 buildings owning dogs, and the average number of owned dogs per household.
- For unowned free-roaming dogs, although they were ownerless, they are usually fed by local feeders, e.g., Buddhist monks and kind aunties [13]. Therefore, these dogs live near public places (G2 or G3 buildings) where local feeders usually provide them food. Since G2 and G3 buildings have no confinable fences, these unowned dogs can roam freely. The numbers of unowned free-roaming dogs residing in G2 and G3 buildings were estimated based on the number of buildings in each category, the proportion of buildings that own dogs, and the average number of unowned free-roaming dogs per dog-owning buildings (Table 1). Based on our assumption, about 91% of unowned free-roaming dogs in Hatyai and 99% of unowned free-roaming dogs in Tepha live in G2 buildings, while the rest live in G3 buildings (Fig 1 (a)).

The word “stray” has been changed to “unowned free-roaming” throughout the manuscript.

**Comment 12**: “*Line 164: delete "then"* ”

**Response:** The paragraph has been thoroughly revised (please see Comment 11).

**Comment 13**: “*Line 165: I suggest to never use "see" before you refer to a table or figure. This is uncommon in scientific papers.*”

**Response:** The manuscript has been thoroughly revised as suggested.

**Comment 14**: “*Line 166: Please state that because of this feeding, they can be assumed to stay around a building that can be classified as their home building (if correct).*”

**Response:** Revised as suggested. The following sentences have been added:

- For unowned free-roaming dogs, although they were ownerless, they are usually fed by local feeders, e.g., Buddhist monks and kind aunties [13]. Therefore, these dogs live near public places (G2 or G3 buildings) where local feeders usually provide them food. Since G2 and G3 buildings have no confinable fences, these unowned dogs can roam freely.

**Comment 15**: “*Line 168: what type of building? Only  G2 or also G3?*”

**Response:** We apologize for any confusion. Both the G2 and G3 buildings have been considered. We calculated the number of dogs in both G2 and G3 buildings. They added up to the total number of unowned free-roaming dogs. To clarify this point, the sentence has been revised, which now reads

- The numbers of unowned free-roaming dogs residing in G2 and G3 buildings were estimated based on the number of buildings in each category, the proportion of buildings that own dogs, and the average number of unowned free-roaming dogs per dog-owning buildings (Table 1).

**Comment 16**: “*Line 170:. I would say that these are not estimations, but assumptions according to table 1. Also, I suggest to report the exact percentage of stray dogs in G2 and G3 buildings based on the assumptions (if I am correct almost 100x more in G2 buildings) already here.*”

**Response:** Revised as suggested. The sentence has been revised, which now reads

- Based on our assumption, about 91% of unowned free-roaming dogs in Hatyai and 99% of unowned free-roaming dogs in Tepha live in G2 buildings, while the rest live in G3 buildings (Fig 1 (a)).

**Comment 17**: “*Line 192: please describe what type of dogs (owned or unowned?) are considered to immigrate and discuss that also the other type can immigrate and think about what effect this might have to not consider the other type of dogs.*”

**Response:** In our study, all imported dogs were assumed to be unowned. To make this clear, the following sentence has been added at the end of the paragraph:

- All imported cases were assumed to be unowned free-roaming dogs.

To investigate a possible effect that might happen due to this assumption, a sensitivity analysis in which an imported dog was assumed to be either an owed dog or an owned free-roaming dog has also been performed. We found that in this case, the likelihood of secondary infection due to the imported dog in Tepha is slightly decreased from 0.03 to 0.02, while that in Hatyai did not change. The following sentences have been added to the first paragraph in the subsection of “Influence of geographical characteristics on the spatiotemporal transmission dynamics of rabies.”

- A sensitivity analysis has also been performed when the imported dog is owned rather than unowned. In this case, we found that the likelihood of secondary infection due to the imported owned dog in Hatyai and Tepha is 0.18 and 0.02, respectively.

**Comment 18-1**: “*Line 200: transmission rate p: indicate that this is probability of bite * probability of transmission given a bite. It is said "model fitting": please explain how this is done. Was the fit done to the rabies observation data? Is so (which is a typical approach by model building), it is expected that the model predictions correlate with the observed data. So this argument cannot be taken as a criteria how good the model represents reality, but only that it well reproduces what is observed. (see also my comment further down for line 384).*”

**Response:** We revised the manuscript as the reviewer suggested. To clarify this point, the following sentences have been added to the end of the second paragraph in the subsection of Model structure:

- To estimate the value of p, a computational search algorithm was employed [15]. Specifically, the model searched for the value of *p* that provides the best fit between the simulation result and the reported cumulative rabies cases. Possible values of *p* that the model can search for are uniformly distributed in the interval [10^-6^, 10^-4^] with 10^-6^ resolution.

We also have removed the sentence both on line 384 and in the abstract.

**Comment 18-2**: “*If p = 1*10-5, then the probability of a bite happening when two dogs encounter is very low, i.e. roughly 2*10-5, e.g. only two times per 10'000 occasions. Is this realistic? Or do I do the maths wrong?*”

**Response:** We apologize that the original name of parameter *p* might be misleading. We, therefore, would like to change its name to “a parameter representing the likelihood that a susceptible dog will be infected by an infectious dog”. In our study, we estimated *p* by fitting our modeling result to the reported data.

Note that since we employed an individual-based approach in our study, we should interpret the force of infection, $\lambda_{i}(t)=\sum_{j=1}^{N_{I}(t)} {pK}_{ij}$, as a probability per unit of time that **each susceptible dog will be infected**. (Please see <https://iopscience.iop.org/article/10.1088/0034-4885/77/2/026602> for more details). To get a rough idea, let us consider a very simple (but unrealistic) situation in which rabies transmission is homogenous. In this case, *K_ij_* will be a constant and does not depend on the geographical locations of dogs. Let us further assume that *K_ij_* = 1, so in this case $\lambda$=$N_{I}p$. If there is only one infectious dog ($N_{I}$ = 1), then $\lambda$= $p$. So, this means that each susceptible dog can be infected with a probability of *p* = 10^-5^ per day. In Hatyai, there are about 207,818 dogs, and about a half of them are unvaccinated, so there would be about one dog infected each day. Similarly, in Tepha, there are 48,142 dogs, so there would be about 0.24 dogs infected each day. We believe that these numbers are reasonable. However, in our study, these numbers can be different from 1 and 0.24 depending on the assumed mean traveling distances and locations of dogs.

**Comment 19**: “*Table 1: please replace public places by G2 to be consistent*”

**Response:** Revised as suggested.

**Comment 20**: “*Line 222: This distance of 1 km a bit comes out of the blue. Why did you pick this number? This is the first time this number if mentioned. I would have expected to describe this already in the population and movement section.”*

**Response:** We aimed to visually illustrate the difference in encountering rates between dogs living in buildings near roads and buildings far away from roads. There is no particular reason why we chose 1 km as an illustration example; however, 1 km is the longest mean traveling distance that a susceptible dog can go (Table 1). To clarify this point, the caption of Figure 1 (d) has been slightly changed, which now reads

- (d) An illustrative example of the unnormalized encountering rate between a rabid dog residing at the centered black point and susceptible dogs living one kilometer apart. The blue and green dots on the map represent the home locations of susceptible dogs that are located near roads and far from roads, respectively. The unnormalized encountering rates are indicated by the colors of the dot circumferences.

**Comment 21**: “*Line 246 : I do not understand this sentence. Also I think it would better fit to the methods section.”*

**Response:** We would like to explain that we measured distances, taken pairwise, between the buildings, similar to finding a distance matrix in mathematics (<https://en.wikipedia.org/wiki/Distance_matrix> ). We then sorted the distance from shortest to longest. The shortest pairwise distance is called the “1^st^ rank of closeness” in our manuscript. To clarify this point in the manuscript, sentences in the second paragraph of the subsection “Geographical characteristics of Hatyai and Tepha districts”, which now read

- We also measured the pairwise distances between a given building and all other buildings and sorted the pairwise distances from shortest to longest. The median pairwise distance with a different rank of closeness (from shortest to longest) is shown in **Fig 3** (c).

We think it might be more straightforward for the readers to describe how we obtained Figure 3 (C ) somewhere around Figure 3 (C ). So, we would like to leave those sentences in that section.

**Comment 22**: “*Fig 3: please add the names of the districts in the graphs a) and b). Also, b) is 10 times more dense than a) Is this correct?”*

**Response:** Yes, the reviewer understands correctly. The scale bar for the building density is 10 times higher in Hatyai than in Tepha. The difference of scales of building density has now been mentioned in the figure caption. Fig 3(a-b) has also been updated following the suggestion, which now reads

Fig 3. Building density and distribution. (a-b) Spatial distribution of building density in Hatyai and Tepha. The color bar indicates the building density in the unit of buildings per square kilometer. Note that the color bar in Hatyai represents 10 times higher building density than in Tepha. The black circles show the centroid points of the building distribution. (c) The median pairwise distance between two buildings with a different rank of closeness (from shortest to longest) in Hatyai (HY) and Tepha (TP) district. The inset illustrates an example of a building arrangement with different ranks of closeness relative to the stared building. (d) The distribution of the shortest building-to-road distance in Hatyai (HY) and Tepha (TP).

**Comment 23**: “*Line 302: Please quickly provide the conclusion what was found in the sensitivity analysis here.”*

**Response:** Revised as suggested. The following sentences have been added at the end of the paragraph:

- A sensitivity analysis of the traveling distance was also performed. We found that when the distances traveled by dogs increase, the spreading pattern of rabies in Hatyai was found to be more dispersed, while the spreading pattern in Tepha is generally not changed (Fig. S3).

**Comment 24**: “*Line 333: I suggest to stick to % and not use the decimal for 0.8.*”

**Response:** Revised as suggested.

**Comment 25**: “*Line 343: 0.33 to 0.78: this is an increase, not a reduction.*”

**Response:** We apologize for the mistake. Actually, it was a reduction, but we mistyped the number. The sentence has been corrected, which now reads

- The likelihood of secondary infection in Hatyai fell from 0.33 to 0.28 (15 %) after a 20% restriction …

**Comment 26**: “*Fig 6: why are the starting points of the likelihood different for each simulation?*”

**Response:** The original Figure 6 did not include the result from the baseline simulation in which reduction fraction = 0, confinement fraction = 0.48, and vaccination coverage = 0.5. Figure 6 has been revised to include those data points and the sensitivity analyses of dog traveling distances on the effectiveness of interventions (please see suggestion 2 from the Editor), which now reads

**Fig 6. Impact of** intervention strategies on **the** likelihood of rabies transmission in Hatyai (a-c) and Tepha (d-f). The likelihood of rabies transmission caused by an imported infected dog under the intervention scenarios of (a, d) reducing the dog population size, (b, e) reducing the fraction of free-roaming dogs, and (c, f) increasing the mass vaccination coverage. A sensitivity analysis where the dog traveling distances are scaled by factors of 0.5 (x0.5) and 2 (x2) was also shown (dashed lines).

**Comment 27**: “*Line 365: I suggest to use "small scale structures" rather than "exact" because for the larger scale models the structures may also have been included exactly.*”

**Response:** Revised as suggested.

**Comment 28**: “*Line 384: which is expected because the incidence data were used to fit the model parameters.*”

**Response:** We agree with the reviewer. That sentence has been removed. The paragraph now reads as follows:

- To test the hypothesis, we simulated and compared the rabies transmission dynamics in Hatyai and Tepha districts. We found that under the same set of epidemiological parameters, the disease incidence rate in Hatyai is higher than in Tepha. Therefore, our simulation results indicated that the difference in the geographical feature distribution in these two districts might be one of the main factors contributing to the difference in the rabies transmission dynamics in these two areas.

**Comment 29**: “*Line 402: I suggest to delete "however"”*

**Response:** The paragraph has been revised (please see Suggestion 1 of the Editor).

**Comment 30**: “*Line 408: Based in the data provided from Chad, why do you come up with 1km max? There is another study (doi: 10.3389/fvets.2021.617900) that would better support your distances.*”

**Response:** Based on this comment and Suggestion 1 of the Editor, the paragraph has been revised and moved to the subsection of “Model structure”, which now reads

- Dog roaming behaviors might be different in various environments. Free-ranging domestic dogs in rural areas in Chad, for example, have home ranges that correspond to a few kilometers of a circular radius [1], whereas free-roaming domestic dogs in Chad, Guatemala, Indonesia, and Uganda move little more than one hundred meters from their home [2]. In the lack of empirical data of dog movement in our study sites, we assumed that free-roaming dogs, i.e., dogs residing in buildings without confinable fences, travel at a mean distance of 500 meters from their homes. Confined owned dogs, i.e., dogs living in the G1 buildings with confinable fences, were assumed to travel around their home territory with a mean distance of 50 meters. Our model assumed that roads would facilitate dog wandering [3-7]; thus, dogs living in buildings near roads could travel further than dogs far from roads. (**Table 1**). However, a sensitivity analysis of the dog's traveling distance was also performed by scaling the mean traveling distances by factors of 0.5 and 2. Note also that although there is a study pointed out that high-traffic roads could be major geographical obstacles to dog roaming [8], the road networks in our study areas are dominated by small local roads (96.65 % for Hatyai and 94.59 % for Tepha, Fig S1), we hence did not consider the possible barrier role of roads in our study.

**Comment 31-1**: “*Line 424: The comparison with the study in Tanzania might be critical, because I guess when you reduce the population in your model by 100, there might be almost no transmission anymore? …”*

**Response:** Thank you to raise this point up. We the paragraph has been revised as shown below

- In this study, we investigated three intervention strategies, i.e., dog population reduction, dog movement restriction, and mass vaccination. Despite dog density matters for rabies persistence, our results highlighted that reducing the dog population density could diminish the likelihood of rabies transmission. However, if the reduction proportion is inadequately high to clear susceptible dogs within the traveling length of rabid dogs, transmission is still feasible. For example, in Hatyai, where dog density is 243 dogs/km^2^, culling half of the population still leaves 381 dogs within a one-kilometer roaming radius of a rabid dog. Our findings are also in line with several other research studies, which show that diminishing the dog population in a moderate way is unlikely to have a beneficial impact on rabies control [16-19]. This indicated that implementing only culling strategies might not be effective enough to eliminate rabies.

**Comment 31-2**: “… *Also, I am wondering whether these dogs living in lower densities do not roam further.”*

**Response:** It is possible that the lower density of dogs might cause an increase in the dog's traveling distance as a result of less territorial overlap between dog packs, especially stray dogs. However, there are other plausible factors that are involved in the range of dog roaming. For example, food supply and shelters are essential factors that may cause dogs to stay near food and prefer not to leave their territory for foraging.

**Comment 32**: “*Line 449: not only modelling studies have limitations, but in all sorts of studies.*”

**Response:** We have deleted the word “modeling”. The sentence now reads

- Like other studies, there were some limitations in our study.

**Comment 33**: “*Line 455: "especially for rabid dogs…" This opens a substantial of follow-up question: you considered healthy dogs in the model, how much do the results hold true when dogs become rabid?*”

**Response:** The term "random movement" has been used to describe the movement of both healthy and rabid animals. For example, diffusion terms have been added to differential equations to model spatial spread of rabid foxes (<https://doi.org/10.1098/rspb.1986.0078>), rabid bats (<https://doi.org/10.1016/j.jtbi.2012.08.033>), and dogs (doi: 10.11948/2012008).

**Comment 34**: “*Line 458: and what is the consequence of this? Do you think this estimation is appropriate? Can it be compared with other studies in Thailand?*”

**Response:** According to a recent study in Thailand (Weerapong Thanapongtharm et al. Front. Vet. Sci., 21 December 2021: <https://doi.org/10.3389/fvets.2021.790701>), the distribution of owned dogs is primarily influenced by residential areas, human population density, and proximity to roads, whereas community, human population density, and proximity to temples were the strongest predictors of ownerless dog distribution. We do believe that our estimates of the number of owned dogs based on the number of residential buildings and the number of unowned dogs based on the number of places where unowned dogs typically reside, such as temples, are reasonable.

In the article, it was also discovered that the urban areas showed a higher density of dogs compared to the rural areas. For example, in Chiang Mai province in northern Thailand, the estimated dog density of Mueang Chiang Mai district as the urban area was 184 heads/km^2^, while the estimated dog density of Fang district in rural areas was 29 heads/km^2^. In comparison to our estimates, 243 heads/km^2^ in Hatyai and 43 heads/km^2^ in Tepha appear to be acceptable, though the density in Hatyai is slightly higher.

The last paragraph has been revised, which now reads

- Like other studies, there were some limitations in our study. Firstly, we only consider the facilitator function of roads. Although our study sites are dominated by small local roads, some high-traffic roads could be geographical obstacles to dog roaming [8]. In addition, since dog movement pattern has not been exactly known, especially for rabid dogs, we assumed that they move randomly, and hence their spatial dispersal could be described by a Gaussian function. Roaming parameters were also assumed using a range of values from the literature. Third, as the exact dog population size was not available in Thailand, we estimated the dog population size based on the type and building density, the fraction of buildings with dogs, and the average number of dogs per building with dogs (Table 1). The owing fraction may vary from place to place. Lastly, although dog vaccination strategy was taken into consideration, dogs were vaccinated without regard for geographical priority. This may not reflect the real-world situation in which control measures are usually applied where a rabies outbreak appears.

**Comment 35**: “*I think REF 33 and 37 are the same.*”

**Response:** We apologize for the mistake. The citations have been corrected.

**References**

1.	Wilson-Aggarwal, J.K., et al., *High-resolution contact networks of free-ranging domestic dogs Canis familiaris and implications for transmission of infection.* PLOS Neglected Tropical Diseases, 2019. **13**(7): p. e0007565.

2.	Warembourg, C., et al., *Comparative Study of Free-Roaming Domestic Dog Management and Roaming Behavior Across Four Countries: Chad, Guatemala, Indonesia, and Uganda.* Frontiers in Veterinary Science, 2021. **8**(96).

3.	Brunker, K., et al., *Landscape attributes governing local transmission of an endemic zoonosis: Rabies virus in domestic dogs.* Mol Ecol, 2018. **27**(3): p. 773-788.

4.	Brunker, K., et al., *Integrating the landscape epidemiology and genetics of RNA viruses: rabies in domestic dogs as a model.* Parasitology, 2012. **139**(14): p. 1899-1913.

5.	Hassine, T.B., et al., *Rabies in Tunisia: A spatio-temporal analysis in the region of CapBon-Nabeul.* Acta Tropica, 2021. **216**: p. 105822.

6.	Numminen, E. and A.-L. Laine, *The spread of a wild plant pathogen is driven by the road network.* PLOS Computational Biology, 2020. **16**(3): p. e1007703.

7.	Fooks, A.R. and A.C. Jackson, *Rabies: Scientific Basis of the Disease and Its Management*. 2020: Elsevier Science & Technology.

8.	Laager, M., et al., *A metapopulation model of dog rabies transmission in N’Djamena, Chad.* Journal of Theoretical Biology, 2019. **462**: p. 408-417.

9.	Michell, A.R., *Longevit of British breeds of dog and its relationships with-sex, size, cardiovascular variables and disease.* Veterinary Record, 1999. **145**(22): p. 625-629.

10.	Hampson, K., et al., *Transmission Dynamics and Prospects for the Elimination of Canine Rabies.* PLOS Biology, 2009. **7**(3): p. e1000053.

11.	S. Premashthira, S.S., W. Thanapongtharm, O. Sagaraseranee, W. Thichumpa, C. Sararat and	A. Wiratsudakul, *The impact of socioeconomic factors on knowledge, attitudes, and practices of dog owners on dog rabies control in Thailand.* Front. Vet. Sci., 2021.

12.	Kongkaew, W., et al., *Vaccination coverage and epidemiological parameters of the owned-dog population in Thungsong District, Thailand.* Prev Vet Med, 2004. **65**(1-2): p. 105-15.

13.	Komol, P., et al., *The spread of rabies among dogs in Pranburi district, Thailand: a metapopulation modeling approach.* Frontiers in veterinary science, 2020. **7**.

14.	Saltelli, A., et al., *Five ways to ensure that models serve society: a manifesto*. 2020, Nature Publishing Group.

15.	Suparit, P., A. Wiratsudakul, and C. Modchang, *A mathematical model for Zika virus transmission dynamics with a time-dependent mosquito biting rate.* Theoretical Biology and Medical Modelling, 2018. **15**(1): p. 1-11.

16.	Townsend, S.E., et al., *Designing Programs for Eliminating Canine Rabies from Islands: Bali, Indonesia as a Case Study.* PLOS Neglected Tropical Diseases, 2013. **7**(8): p. e2372.

17.	Windiyaningsih, C., et al., *The rabies epidemic on Flores Island, Indonesia (1998-2003).* J Med Assoc Thai, 2004. **87**(11): p. 1389-93.

18.	Putra, A.A., et al., *Response to a rabies epidemic, Bali, Indonesia, 2008-2011.* Emerg Infect Dis, 2013. **19**(4): p. 648-51.

19.	Wera, E., et al., *Costs of Rabies Control: An Economic Calculation Method Applied to Flores Island.* PLOS ONE, 2013. **8**(12): p. e83654.
